# CD13 is a critical regulator of cell–cell fusion in osteoclastogenesis

**DOI:** 10.1038/s41598-021-90271-x

**Published:** 2021-05-24

**Authors:** Mallika Ghosh, Tomislav Kelava, Ivana Vrhovac Madunic, Ivo Kalajzic, Linda H. Shapiro

**Affiliations:** 1 Center for Vascular Biology, University of Connecticut Medical School, Farmington, CT 06030 USA; 2 Center for Regenerative Medicine and Skeletal Development, University of Connecticut Dental School, Farmington, CT 06030 USA

**Keywords:** Cell biology, Immunology

## Abstract

The transmembrane aminopeptidase CD13 is highly expressed in cells of the myeloid lineage, regulates dynamin-dependent receptor endocytosis and recycling and is a necessary component of actin cytoskeletal organization. Here, we show that CD13-deficient mice present a low bone density phenotype with increased numbers of osteoclasts per bone surface, but display a normal distribution of osteoclast progenitor populations in the bone marrow and periphery. In addition, the bone formation and mineral apposition rates are similar between genotypes, indicating a defect in osteoclast-specific function in vivo. Lack of CD13 led to exaggerated in vitro osteoclastogenesis as indicated by significantly enhanced fusion of bone marrow-derived multinucleated osteoclasts in the presence of M-CSF and RANKL, resulting in abnormally large cells containing remarkably high numbers of nuclei. Mechanistically, while expression levels of the fusion-regulatory proteins dynamin and DC-STAMP1 must be downregulated for fusion to proceed, these are aberrantly sustained at high levels even in CD13-deficient mature multi-nucleated osteoclasts. Further, the stability of fusion-promoting proteins is maintained in the absence of CD13, implicating CD13 in protein turnover mechanisms. Together, we conclude that CD13 may regulate cell–cell fusion by controlling the expression and localization of key fusion regulatory proteins that are critical for osteoclast fusion.

## Introduction

Osteoclastogenesis is a critical process for skeletal growth and development that is tightly regulated by differentiation of myeloid progenitor cells into osteoclasts, which are specialized bone marrow (BM)-derived cells whose major function is bone resorption^1^. This process is responsible for bone modeling and remodeling that ultimately translates into maintenance of bone integrity, skeletal growth and repair^2,3^. Deregulation of the balance between bone destruction and synthesis leads to dramatic outcomes^4,5^. Loss of osteoclast (OC) generation/function gives rise to elevated bone formation, resulting in osteopetrosis, characterized by high bone mass and growth impairment. Alternatively, gain of OC generation/function results in exacerbated bone degradation that, without equivalent coupling to bone formation, leads to osteoporosis, characterized by low bone mass, bone weakness and high predisposition to fractures with poor healing progression. OCs form from committed monocyte progenitors via initial signals provided by two key BM-derived cytokines: macrophage colony stimulating factor (M-CSF) and receptor activator of NFκB ligand (RANKL), initiating a highly-organized program of commitment towards terminal differentiation and function^6–8^. This program requires the precise regulation of the expression of cell–cell fusion proteins that is critical to the generation of functionally active OCs. Despite our current knowledge of events leading to the formation of OCs, understanding the regulation of these processes in homeostatic and pathological conditions is unclear^9–11^.

CD13 is a transmembrane metalloprotease widely expressed in all cells of the myeloid lineage, activated endothelial cells, hematopoietic progenitor and stem cells^12–16^. Previous studies have implicated CD13 in vasculogenesis, tumor cell invasion, inflammatory trafficking and as a receptor for a strain of human coronavirus^17–19^. Our novel observations have clearly shown that independent of its peptidase activity, CD13 is a critical regulator that assembles the molecular machinery enabling diverse cellular processes such as cell–cell adhesion, migration, membrane organization, dynamin-mediated receptor endocytosis and recycling of cell surface proteins^14,15,20–22^. Taken together, CD13 regulates many of the activities that have been described to be critical for osteoclastogenesis and cell–cell fusion. In the current study, we demonstrate that despite a relatively normal distribution of hematopoietic components in bone marrow and periphery^12^, bone mass in CD13^KO^ mice is reduced and OC number per bone surface area is increased while bone formation parameters are normal, indicating that in the absence of CD13, osteoclastogenesis is perturbed without affecting osteoblast function. In addition, the in vitro cytokine-mediated induction of flow-sorted BM-derived CD13-deficient OC progenitors generated increased numbers of OCs that were considerably larger in size, contained many more nuclei and resorbed bone much more efficiently than those from wild type progenitors. Consistent with the primary murine OC data, human osteoclast-like cells generated from CRISPR- engineered CD13^KO^ U937 cells led to an increase in multinucleated, TRAP+, functional OC compared to the control cells, confirming that CD13 modulates osteoclastogenesis. Furthermore, we demonstrated that while the expression of certain fusion proteins is typically downregulated in mature osteoclasts post-fusion^23^, it is abnormally preserved via post-transcriptional mechanisms in osteoclasts lacking CD13. We have shown that CD13 is a mediator of homotypic cell interaction and a regulator of molecular events defining cell membrane organization, fluidity and movement, all processes critical to cell–cell fusion^14,15,20^. We hypothesize that CD13 is a negative regulator of cell–cell fusion in osteoclastogenesis and potentially, a universal modulator of membrane fusion and thus is a novel target for therapeutic intervention in pathological conditions mediated by defects in cell–cell fusion.

## Results

### Bone density is reduced in CD13^KO^ mice in vivo

**Figure 1:**
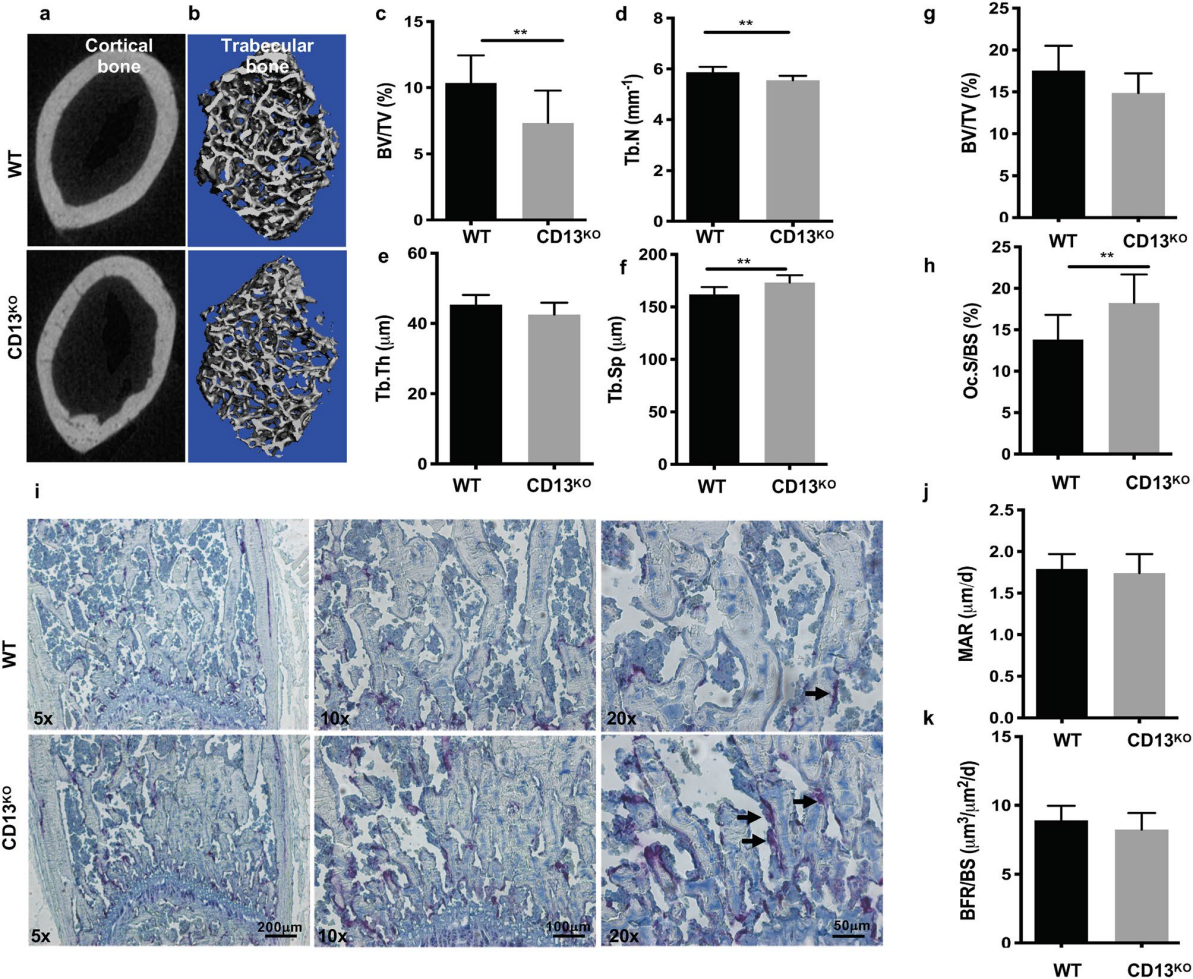
Loss of CD13 leads to reduced bone volume and increased number of osteoclasts in 8–10 week old CD13-deficient mice. µCT reconstruction of cortical (**a**) and trabecular bone (**b**). (**c**–**f**) Bone morphometry by µCT analysis; (**c**) BV/TV(%); Bone volume/Tissue volume, (**d**) Trabecular number, (**e**) Trabecular Thickness, (**f**) Trabecular Separation. (**g**–**i**) Histomorphometric analysis of femurs of WT and CD13^KO^. Histology of femurs with (**g**), BV/TV(%), (**h**) Oc.S/BS; % OCs per bone surface (**i**), histochemical detection of TRAP^+^ osteoclasts (purple, indicated by the arrow). (**j**) Mineral apposition rate (MAR), (**k**) Bone formation rate (BFR/BS) measured by Osteomeasure software (OsteoMetrics, Decatur, USA) (https://www.osteometrics.com). All samples were scanned, reconstructed, and analyzed in a Scanco µCT40 running Evaluation Program V6.6 (http://www.scanco.ch/en/systems-solutions/software.html). Histochemical analysis of TRAP^+^ osteoclasts in bone sections were imaged with Zeiss fluorescence inverted microscope and analyzed by using Zeiss Zen 2.0 Pro blue edition software (https://www.zeiss.com/content/dam/Microscopy/Downloads/Pdf/FAQs/zen2-blue-edition_installation-guide.pdf). Scale bars-×5; 200 µm, ×10; 100 µm, ×20; 50 µm. Data represents ± SD. (N = 6; WT, N = 7; CD13^KO^. **p < 0.01).

Based on the notion that the highly sustained expression of CD13 in all cells of the myeloid lineage reflects its important role in myeloid cell biology, we and others have demonstrated that it contributes to many fundamental cellular processes that impact myeloid cell function in various tissues. These studies prompted our current focus on the myeloid cells of the bone, the osteoclasts. We initially examined the effect of a global loss of CD13 on the phenotype of developing bone. Analysis of bone micro-architecture and function in the cortical and trabecular bone isolated from 8 to 10 week old WT and CD13^KO^ mice by μ-CT revealed that the femur cortical and trabecular bone density and thickness in CD13^KO^ mice is reduced compared to WT animals (Fig. 1a,b). Reduced bone volume/total volume (BV/TV, WT vs. CD13^KO^; 10.4 vs. 7.3, Fig. 1c) and Trabecular number (Tb.N; Fig. 1d), comparable trabecular thickness (Tb.Th) (Fig. 1e) with increased trabecular spacing (Tb.Sp; Fig. 1f) was observed in the absence of CD13. Similarly, bone histomorphometric analysis indicated a trend in reduction in BV/TV (Fig. 1g) with an increased number of osteoclasts per bone surface (Oc.S/BS, WT vs. CD13^KO^; 13.8 vs. 18.3, Fig. 1h). In addition, histochemical analysis showed that these osteoclasts were TRAP^+^ (in purple) in CD13^KO^ femurs compared to their wildtype counterparts (Fig. 1i). Bone remodeling is tightly controlled by the coordinated action of bone forming osteoblasts and bone degrading osteoclasts whereby an increase in bone resorption with no change in formation can lead to loss of bone density. To investigate potential underlying causes of CD13-dependent loss of bone density in CD13^KO^ mice, we evaluated bone formation in vivo in young mice. Importantly, both the mineral apposition rate (MAR, measurement of the linear rate of new bone deposition), (Fig. 1j) and dynamic bone formation rate (BFR/BS), (Fig. 1k) were unchanged in CD13 deficient mice. These data demonstrated that the overall bone loss in CD13-deficient mice is a result of enhanced osteoclast number and activity while bone formation and osteoblast function is unaltered.

### Defects in bone structure are exaggerated in CD13-deficient aged mice

**Figure 2:**
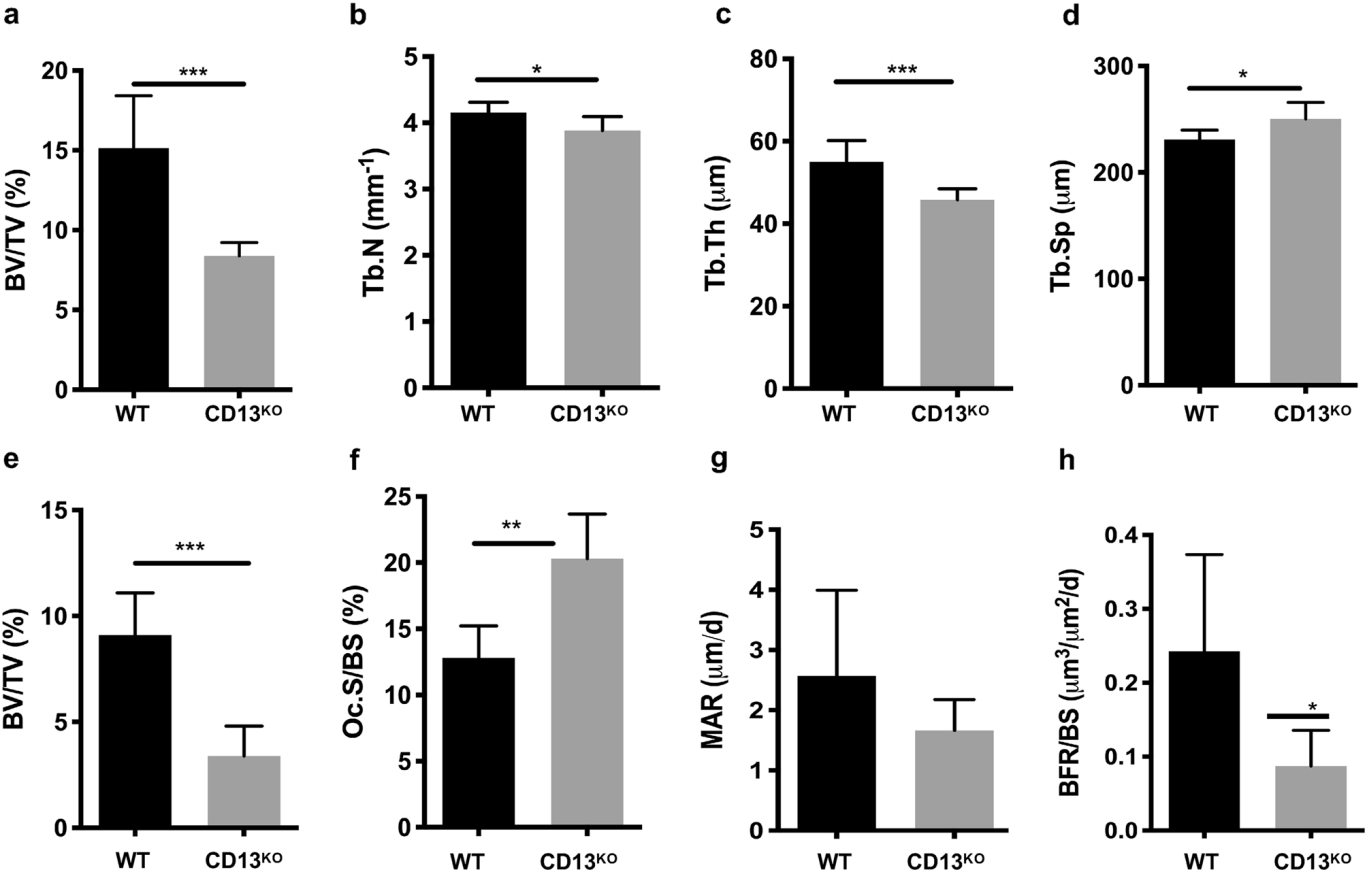
CD13 deficiency leads to marked reduction in trabecular bone volume and structure in 18–25 week old aged mice. (**a**–**d**) Bone morphometry by µCT analysis; (**a**), BV/TV(%); Bone volume/Tissue volume (**b**), Trabecular number, (**c**) Trabecular Thickness (**d**) Trabecular Separation. (**e–h**) Histomorphometric analysis (**e**), BV/TV(%), (**f**) Oc.S/BS; % OCs per bone surface, (**g**) Mineral apposition rate (MAR), (**h**) Bone formation rate (BFR/BS) measured by Osteomeasure software (OsteoMetrics, Decatur, USA) (https://www.osteometrics.com). All samples were scanned, reconstructed, and analyzed in a Scanco µCT40 running Evaluation Program V6.6 (http://www.scanco.ch/en/systems-solutions/software.html). Data represents ± SD. (N = 6; WT and N = 7; CD13^KO^. ***p < 0.001, **p < 0.01, *p < 0.05).

Next, we investigated if the steady state phenotype of lower bone mass observed in CD13 deficient young mice is amplified during aging. Indeed, μCT and histomorphomtery indicated a significant reduction in BV/TV (WT vs. CD13^KO^; 15.0 vs. 7.5; Fig. 2a and WT vs. CD13^KO^; 9.0 vs. 3.4; Fig. 2e), trabecular number (Fig. 2b) and thickness (Fig. 2c), with a concomitant increase in trabecular spacing (Fig. 2d). Finally, the increase in osteoclast numbers (Oc.S/BS, WT vs. CD13^KO^; 12.8 vs. 20.3; Fig. 2f) in CD13-deficient aged mice compared to the wildtype counterpart was sustained. However, over time, while the MAR (Fig. 2g) was unaffected between genotypes in the aged mice, the BFR/BS (Fig. 2h) was significantly diminished in older CD13^KO^ animals, indicating that CD13 also impacts osteoblast function long-term, which may contribute to the accelerated bone loss in CD13^KO^ aged mice (Fig. 2a,e). Together, these data strongly suggested a defect in bone structure in the absence of CD13 that is sustained over time, supporting a contribution of CD13 to homeostatic bone remodeling in vivo.

### In vitro osteoclastogenesis is exaggerated in CD13^KO^ cells

**Figure 3:**
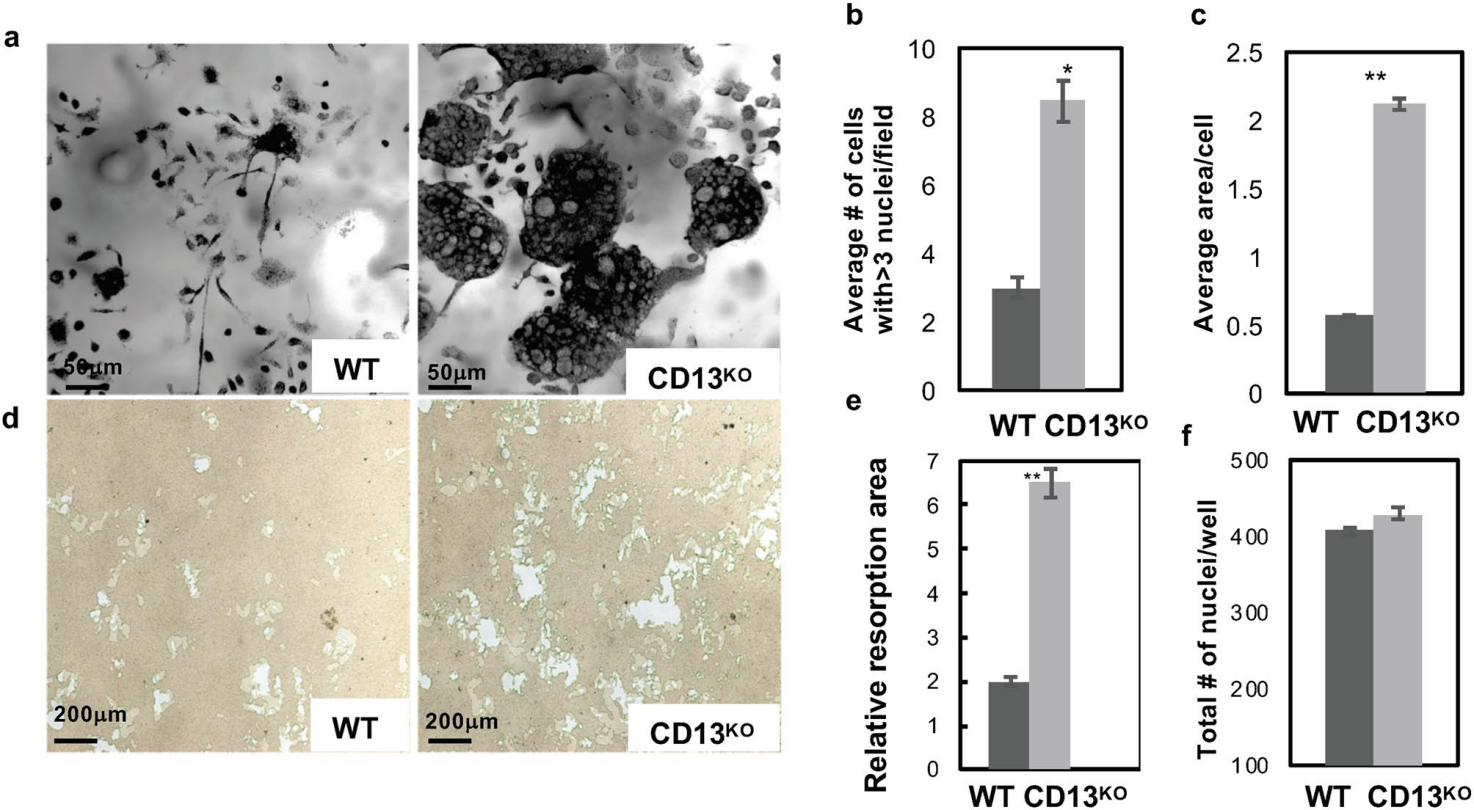
TRAP + multinucleated OCs are increased in the absence of CD13 in vitro. (**a**) Primary murine BM- derived osteoclast progenitor cells grown on bovine cortical slices in presence of M-CSF and RANKL led to increased OCs size and number of nuclei/OC in CD13^KO^ cells compared to WT at d5. (**b**) Average # of cells with > 3 nuclei/field and (**c**) Average cell area per OCs in CD13^KO^ are significantly larger than the WT cells. (**d**) Area of resorption is significantly higher in CD13^KO^ than WT OCs grown on osteoplates for d10 by phase contrast imaging. Osteoclasts on cortical slices and cluster of pits formed were imaged using a light microscope (Olympus Scientific), using Olympus cellSens Dimension V0118 software (Olympus Scientific) (https://www.olympus-lifescience.com/en/software/cellsens/) and the area of resorption was quantified by Image J (https://imagej.nih.gov/ij/) (**e**) and total number of nuclei/well of both genotypes (**f**) are shown. Scale bars-(**a**) 50 µm; (**d**) 200 µm. Data represents ± SEM of three independent experiments. N = 6/genotype, **p < 0.01, *p < 0.05.

**Figure 4:**
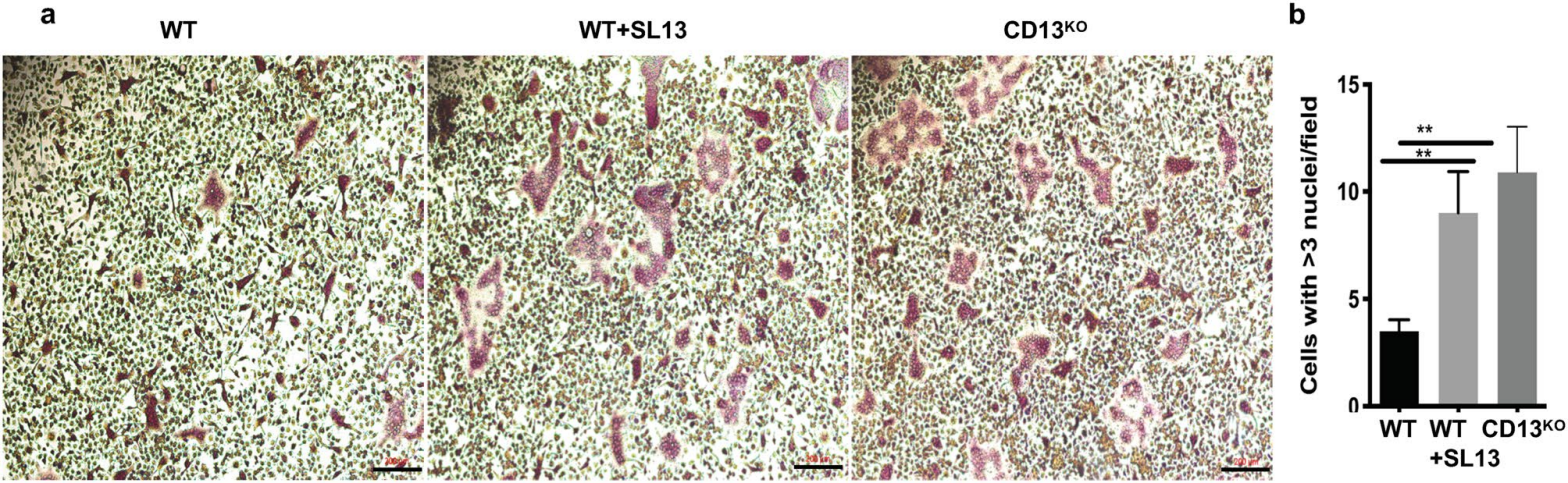
Treatment with CD13 blocking Ab leads to increased multinucleated OC formation in WT cells. (**a**). TRAP staining of WT, WT + SL13 and CD13^KO^ BM-derived osteoclast progenitor cells grown in presence of M-CSF and RANKL after 5d on plastic are shown in “red” color. (**b**). Quantification of Trap^+^ OC with > 3 nuclei/cell. TRAP^+^ osteoclasts were imaged with Zeiss fluorescence inverted microscope and analyzed by using Zeiss Zen 2.0 Pro blue edition software (https://www.zeiss.com/content/dam/Microscopy/Downloads/Pdf/FAQs/zen2-blue-edition_installation-guide.pdf). Scale bar; 200 µm. Data represents ± SEM of three independent experiments. N = 3/genotype, **p < 0.01. Magnification ×5.

The previous data suggested that CD13 may mediate remodeling predominantly at the level of the osteoclasts. Stimulation of flow-sorted monocyte lineage-committed hematopoietic progenitors with two principal BM cytokines, M-CSF and RANKL, triggers the expression of molecules involved in cell–cell fusion and functional bone resorption^6–8^ and the generation of multinucleated osteoclasts. Flow sorted, BM-derived OCP (CD3^−^ B220^−^ NK1.1^−^ CD11b^lo/−^ CD115^hi^ Ly6G^+^, Supplementary Fig. S1) were differentiated to mature OC in the presence of recombinant M-CSF and RANKL and stained with TRAP. Analysis of these cultures revealed significant increases in the number of osteoclasts containing > 3 nuclei per field (2.8-fold) and the average OC size (fourfold) in CD13^KO^ cells grown on bovine cortical bone slices or plastic (Figs. 3a–c, 4) compared to those generated from WT progenitors. These differences were evident by d5, suggesting that the lack of CD13 accelerates OC fusion and multinucleation but not OCP proliferation rates, as the cell density (total number of nuclei/dish) was not significantly different between genotypes (Fig. 3f). In agreement with amplified osteoclastogenesis in vivo in CD13-deficient mice, in vitro multinucleated OC formation (Supplementary Fig. S2a–c) was significantly elevated in OCs generated from CD13^KO^ aged BM. Similarly, spleen-derived CD13^KO^ cells produced OCs of larger area (threefold), more cells with > 3 nuclei (2.5-fold) and an increased number of nuclei per cell (threefold) compared to WT (Supplementary Fig. S3). To assess OC bone resorptive capacity, we utilized a 24-well Osteo assay plate, (Corning), coated with synthetic bone mimetic that allows measurement of in vitro osteoclast activity. We plated WT and CD13^KO^ flow-sorted BM-derived OCP on Osteo assay plates in the presence of recombinant M-CSF and RANKL and allowed them to mature to OC over time. At d10, OCs were removed, individual or multiple resorption pit areas were imaged and the area of resorption quantified by ImageJ. As expected, the increase in OC nuclei/cell positively correlates with resorption where CD13^KO^ OCs showed increased resorption area (threefold) compared to WT, confirming that the elevated fusion in CD13^KO^ mice translates into exaggerated functional activity in both young (Fig. 3d,e) and aged (Supplementary Fig. S2d,e) mice. Importantly, WT cells treated with CD13 blocking antibody (SL13; 1 μg/ml) prior to fusion in the presence of M-CSF and RANKL led to a significant increase (threefold) in multinucleated OC with greater than 3 nuclei per cell which was analogous to the CD13-deficient OCs, illustrating that blocking CD13 as well as its absence led to accelerated fusion (Fig. 4a,b) and confirming the CD13 specificity of osteoclast fusion phenotype.

### Osteoclast progenitors with osteoclastogenic potential are similar in WT and CD13^KO^ bone marrow and periphery

Previously we have shown that the distribution of the hematopoietic population comprised of early hematopoietic progenitors, myelo-erythroid progenitors, and granulocyte macrophage progenitors in CD13^KO^ mice were similar to wildtype animals^12^. Cells that can generate bone-resorbing osteoclasts reside in both BM and peripheral hematopoietic organs^24^. To determine if differences in osteoclast progenitor (OCP) frequency are responsible for the loss of bone mass in the absence of CD13, we analyzed the distribution of primary OCP in both the BM microenvironment and spleen in WT and CD13^KO^ mice. Flow cytometric analysis (Supplementary Fig. S4) revealed that the OCP profile indicated by CD3^−^, B220^−^, NK1.1^−^, CD11b^−/lo^, CD115^+^, CD117^+^ in the BM (a, WT vs. CD13^KO^; 1.7 vs. 1.95) and CD3^-^, B220^-^, NK1.1^-^, CD11b^+^, Ly6G^-^, Ly6C^+^, CD115^+^ in spleen (b, WT vs. CD13^KO^; 0.24 vs. 0.238)^24^ and common myeloid progenitor population indicated by lin^−^ c-kit^+^ Sca-1^−^ CD34^+^ in the BM (**c**, WT vs. CD13^KO^; 0.99 vs. 0.88) is similar between genotypes, indicating that the absence of CD13 does not change the intrinsic differentiation potential of myeloid cells to osteoclast progenitors.

### Osteoclast-like cell formation is enhanced in U937 cells expressing CD13 CRISPR

**Figure 5:**
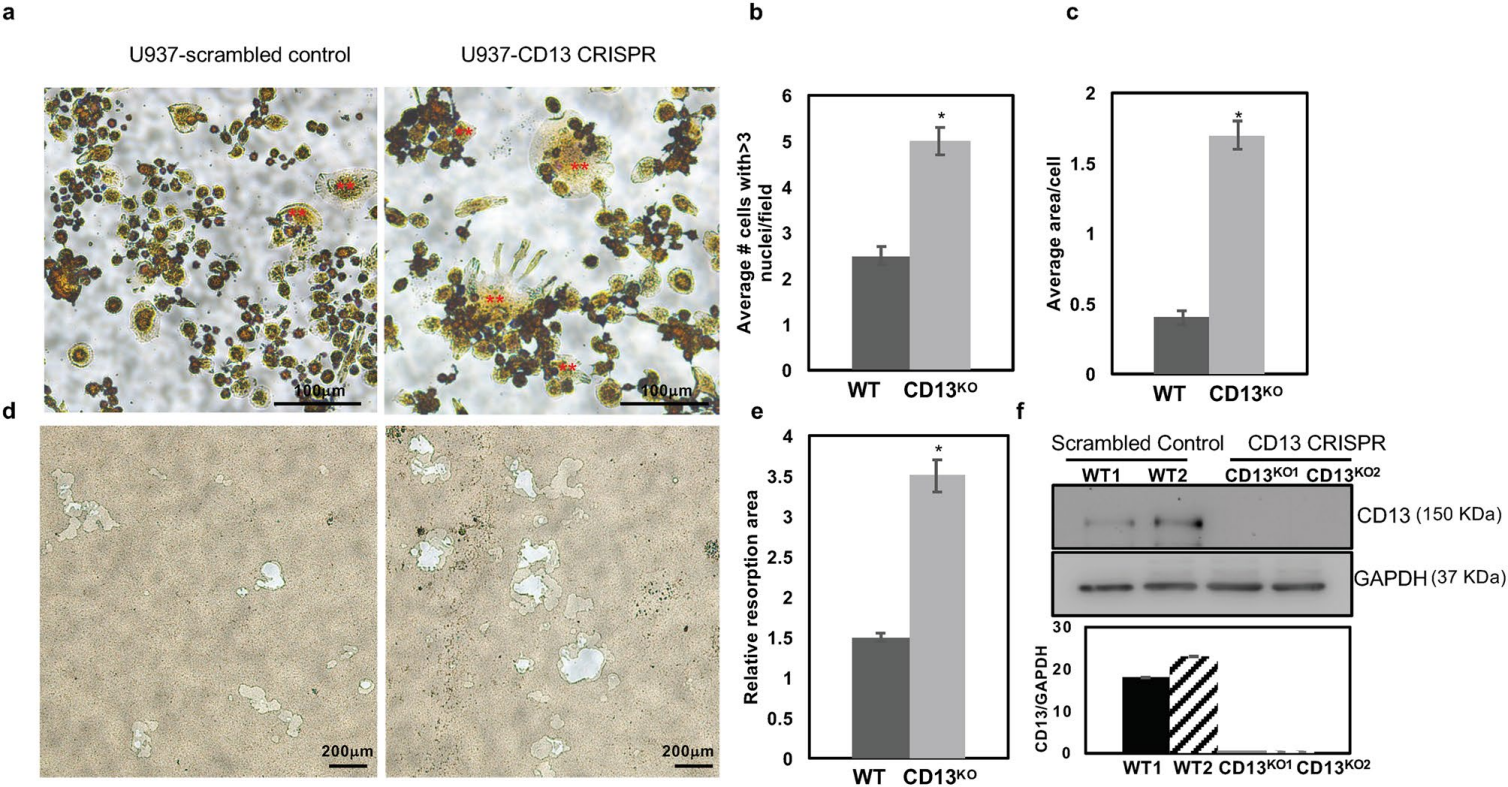
Increased Multinucleated OC formation in U937 cells expressing CD13 CRISPR. (**a**). TRAP staining of U937 expressing scrambled control or CD13 CRISPR grown in presence of PMA for 3 days followed by M-CSF and RANKL for 10 d indicated increased size and number of multinucleated OCs in absence of CD13 compared to WT. (**b**) Multinucleated OC (**) with greater than 3 nuclei per cell and (**c**), average area of OC. (**d**). Phase contrast imaging of area of resorption in U937 cells expressing CD13 CRISPR or scrambled control grown in presence of PMA followed by M-CSF and RANKL for 17 d on osteoplates. TRAP^+^ osteoclasts were imaged with Zeiss fluorescence inverted microscope and analyzed by using Zeiss Zen 2.0 Pro blue edition software (https://www.zeiss.com/content/dam/Microscopy/Downloads/Pdf/FAQs/zen2-blue-edition_installation-guide.pdf). Area of resorption was imaged using a light microscope (Olympus Scientific), using Olympus cellSens Dimension V0118 software (Olympus Scientific) (https://www.olympus-lifescience.com/en/software/cellsens/) quantified by Image J (https://imagej.nih.gov/ij/) (**e**). (**f**). Immunoblot analysis of CD13 expression in U937 cells expressing CD13 CRISPR or scrambled control clones. Blots were imaged by ChemiDoc Imaging system version 3.0.1 (https://www.bio-rad.com/en-us/category/chemidoc-imaging-systems?ID=NINJ0Z15) (Biorad). A cropped image is shown, see Supplementary Fig. S6 for full-length blots and cropped replicates. Scale bar-(**a**) 100 µm; (**d**) 200 µm. Data represents ± SEM of three independent experiments. N = 3/genotype, *p < 0.05. Magnification ×10.

To confirm that the effect of loss of CD13 in cell fusion extends across species, we utilized human U937 myeloid cells engineered to delete CD13 or express scrambled wildtype control by CRISPR-based technology (Fig. 5f). Differentiation of U937 cells with PMA^25–27^ for 3 days led to formation of equivalent levels of adherent monocytic cells in both genotypes (data not shown). Subsequent stimulation with human recombinant M-CSF and RANKL led to the aggregation of monocyte-like cells followed by differentiation into large multinucleated TRAP^+^ osteoclast-like cells over time (8-10d) which was significantly accelerated in cells lacking CD13 compared to scrambled controls (Fig. 5a–c). TRAP+, multinucleated CD13^KO^ CRISPR cells exhibited increased resorptive activity when grown in Osteo assay plates for 17 days as indicated by the relative resorption area (Fig. 5d,e), confirming that the cells generated from U937 are indeed functional osteoclasts and that CD13 is a negative regulator of cell–cell fusion in both mouse primary cells (BM and periphery) and a human monocytic cell line.

### Expression of fusion proteins is dysregulated in CD13^KO^ mature osteoclasts

**Figure 6:**
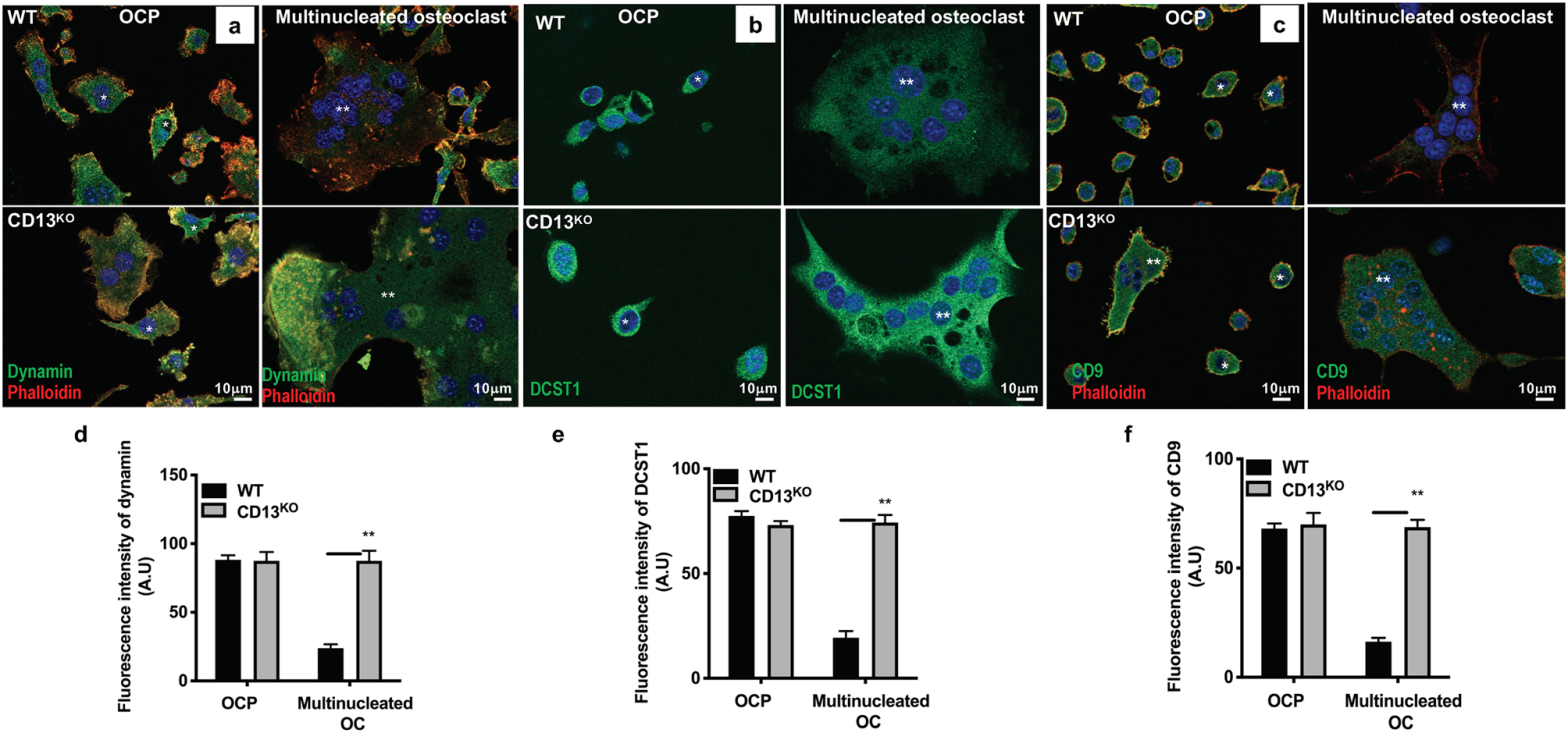
Immunofluorescence analysis of persistent expression of fusion-regulatory proteins in CD13-deficient multinucleated OC. Expression of the fusion regulatory proteins dynamin (**a**,**d**), DCST1 (**b**,**e**) and CD9 (**c**,**f**) is maintained in CD13^KO^ multinucleated osteoclasts but not in WT cells (**). High levels of dynamin co-localize with actin and DCST1 in OCPs (*), imaged using Zeiss LSM 880 confocal fluorescence microscope and analyzed by Zeiss Zen 2.0 Pro blue edition software (https://www.zeiss.com/content/dam/Microscopy/Downloads/Pdf/FAQs/zen2-blue-edition_installation-guide.pdf). Scale bar; 10 µm. Data represents average of three independent experiments. N = 3/genotype. Dynamin, CD9, DCST1; green. Phalloidin; red. Magnification ×63 oil.

**Figure 7:**
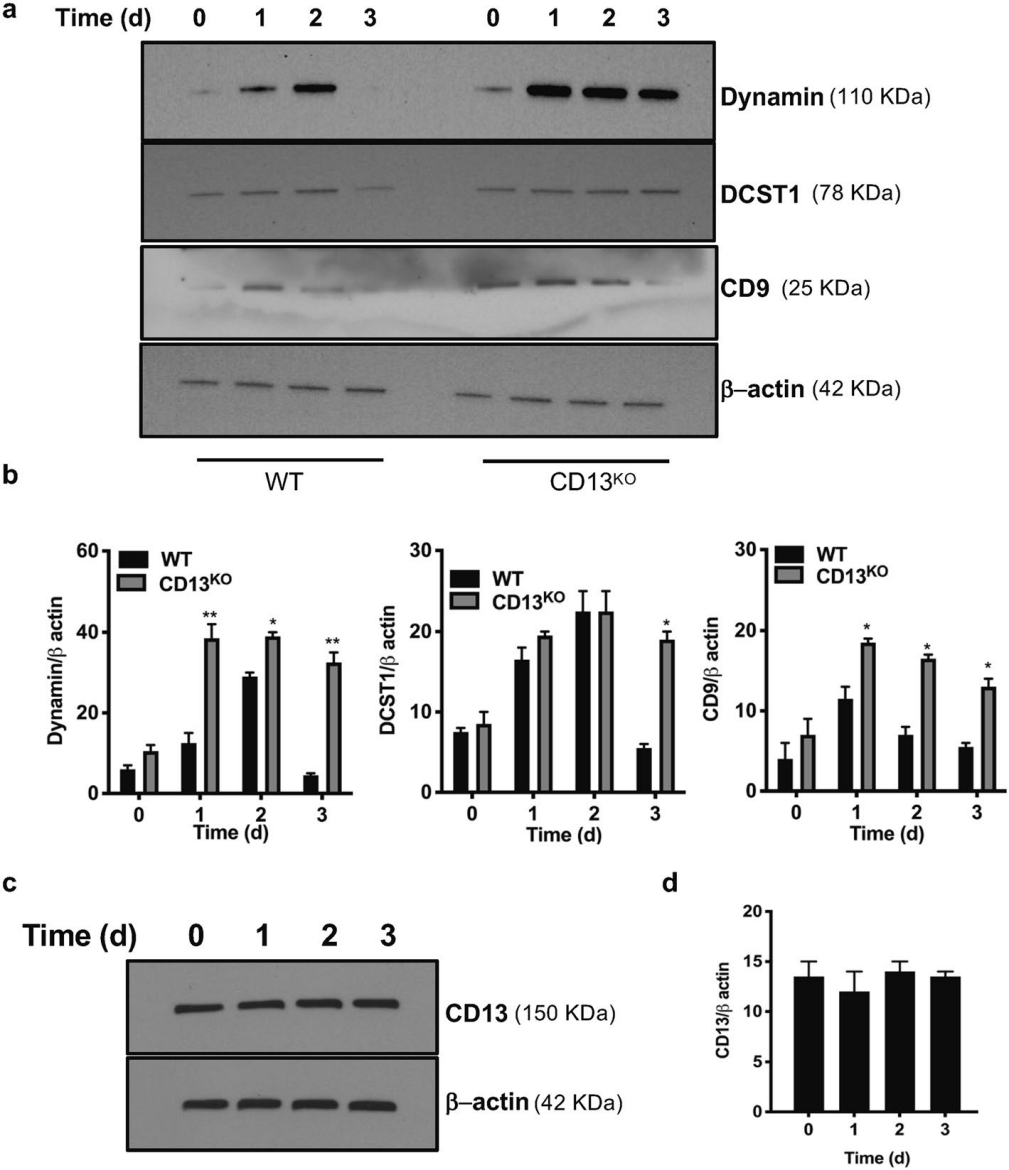
Immunoblot analysis of sustained expression of fusion-regulatory proteins in CD13-deficient OC over time. Expression of the fusion-promoting proteins dynamin, DC-STAMP (DCST1) and CD9 is aberrantly sustained in CD13^KO^ but not in WT multinucleated osteoclasts (**a**,**b**) in presence of M-CSF and RANKL. A cropped image is presented, see Supplementary Fig. S7 for full-length blots and Supplementary Fig. S8 for cropped replicates. (**c**,**d**). CD13 expression is unaltered in BM-derived OCs in response to M-CSF and RANKL stimulation over time. Blots were imaged by ChemiDoc Imaging system version 3.0.1 (https://www.bio-rad.com/en-us/category/chemidoc-imaging-systems?ID=NINJ0Z15) (Biorad). A cropped image is presented, see Supplementary Fig. S9 for full-length blots and cropped replicates. Data represents average of two isolates. N = 3/genotype. **p < 0.01, *p < 0.05.

A number of the molecular mechanisms mediating osteoclast fusion and multinucleation have been elucidated^28^. In particular, the small GTPase dynamin 2 (the major isoform in OC), the “master fusogen” DC-STAMP (DCST1) and the tetraspanins CD9 and CD81 are common and critical regulators of osteoclast fusion^23,29^. Our recent studies have shown that CD13 is a potent negative regulator of dynamin-dependent endocytosis of a variety of receptors^20–22^, suggesting that CD13 may participate in cell fusion by regulating endocytic processes. Indeed, immunofluorescence (Fig. 6) and immunoblot (Fig. 7a,b) analyses of OC lysates derived from flow-sorted WT BM-OCPs demonstrated high levels of dynamin and DC-STAMP expression by 2d-post differentiation, which was subsequently reduced by 3d when cell fusion and maturation into multinucleated WT osteoclasts is complete, as previously reported^23^. However, while dynamin, DC-STAMP and CD9 are highly expressed in CD13^KO^ OCP, rather than being downregulated, this strong expression is maintained in mature multinucleated OCs (Figs. 6, 7a,b), suggesting that CD13 may impact fusion by regulating the levels of these key fusion and/or endocytic molecules critical for OC fusion. In addition, flow cytometry of dynamin, DCST1 and CD9 confirmed persistent surface expression of fusion promoting proteins in multinucleated OC lacking CD13 compared to WT OC (Supplementary Fig. S5a–c). Furthermore, immunoblot analysis of cell lysates obtained from WT BM-derived progenitor cells stimulated with M-CSF and RANKL over 3d indicated that CD13 is highly expressed in myeloid progenitor cells but its expression level is unaltered upon stimulation with M-CSF and RANKL over time (d0-3) (Fig. 7c,d), consistent with CD13 regulating fusion mechanisms independent of RANKL signaling.

**Figure 8:**
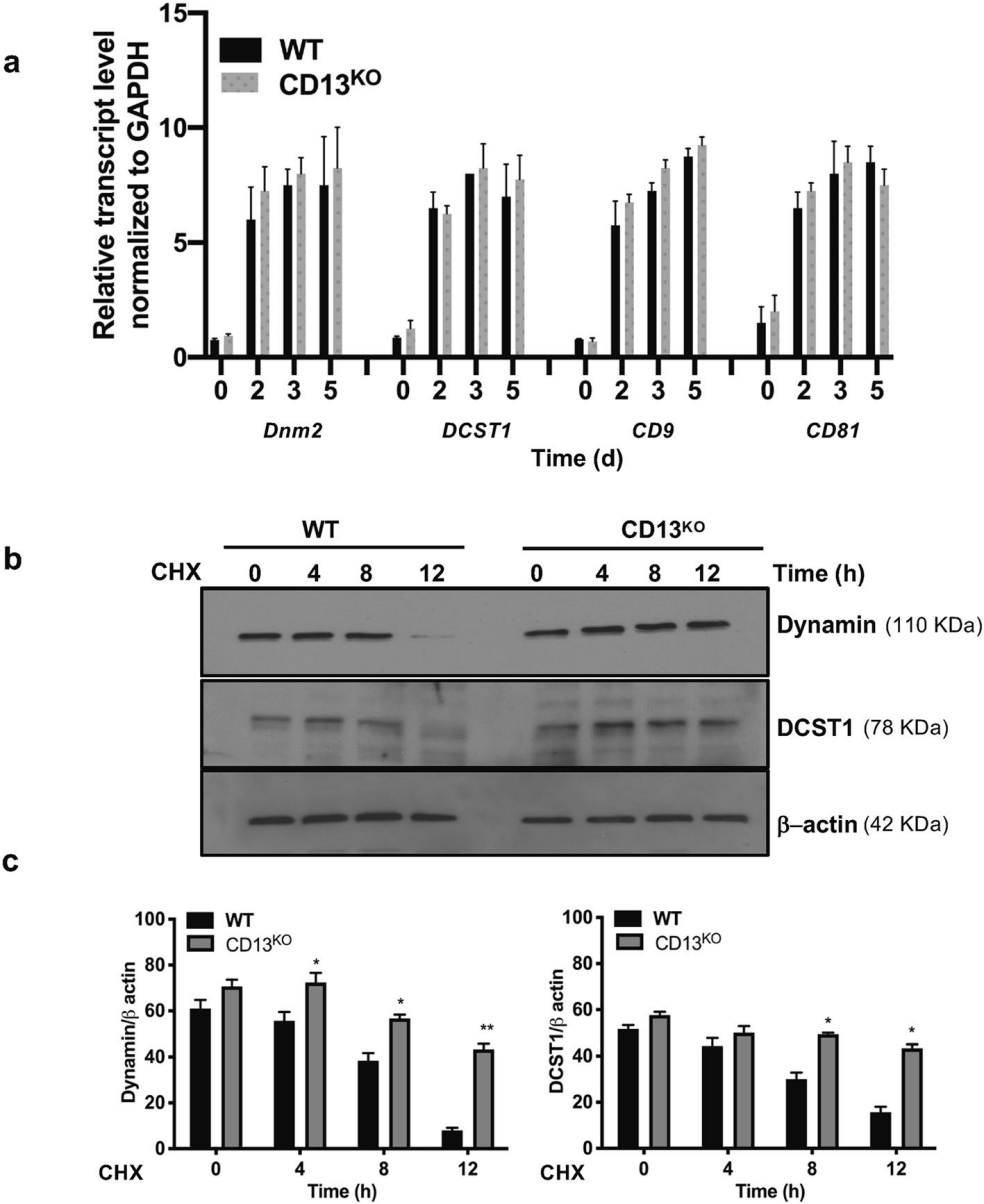
Fusion-regulatory proteins, dynamin and DCST1 are regulated by a CD13-dependent post transcriptional mechanism. (**a**) Quantitative RT-PCR analysis of fusion regulatory transcripts normalized to GAPDH in flow sorted mouse BM cells stimulated with M-CSF and RANKL over indicated time. Expression of the genes regulating osteoclast fusion -dynamin 2 (DNM2), DCST1, CD9 and CD81 are highly induced upon M-CSF and RANKL over time but was equivalent between genotypes. All data was analyzed using CFX Manager version 3.1 ((https://www.bio-rad.com/en-us/sku/1845000-cfx-manager-software?ID=1845000) (Biorad). Data represents average of two independent experiments. N = 3/genotype. (**b**–**c**) Dynamin and DCST1 protein stability are enhanced in absence of CD13. Immunoblot analysis of dynamin and DCST1 of WT and CD13^KO^ BM-derived OC treated with cycloheximide (CHX) for indicated time. Blots were imaged by ChemiDoc Imaging system version 3.0.1 (https://www.bio-rad.com/en-us/category/chemidoc-imaging-systems?ID=NINJ0Z15) (Biorad). A cropped image is presented, see Supplementary Fig. S10 for full-length blots and cropped replicates. Data represents average of two isolates. N = 3/genotype. **p < 0.01, *p < 0.05.

Next, we investigated the overall mechanistic process by which CD13 sustains expression levels of dynamin, DCST1 and tetraspanins. Quantitative RT-PCR analysis demonstrated that transcript levels for *DNM2*, *DCST1* and tetraspanins *CD9* and *CD81* were similar in BM progenitor cells stimulated with M-CSF + RANKL over 0-5d, indicating that CD13 does not regulate transcription levels of these proteins (Fig. 8a). To explore if CD13 limits the stability of fusion-regulatory proteins in WT cells, BM-derived OCP were grown in the presence of M-CSF + RANKL and treated with cycloheximide (100 μg/ml) to inhibit new protein synthesis. While dynamin and DCST1 protein expression in CD13^KO^ OC remained stable over 8–12 h, loss of dynamin and DCST1 protein expression occurred by 4–8 h in WT OC, indicating that CD13 controls fusion-regulatory protein turnover and stability (Fig. 8b,c).

## Discussion

The fusion of plasma membranes is essential to and indispensable for many physiologic processes such as fertilization through sperm/egg fusion^30^, muscular development through myoblast fusion^31^, skeletal development and maintenance of skeletal integrity through formation of osteoclasts and control of certain viral infections and spreading through the formation of macrophage giant cells (MGC)^32^. Thus, this important biological process directly defines the course of many pathological processes including infertility, skeletal defects (osteoporosis and osteopetrosis), failure of skeletal repair, failure to maintain prosthetic implants as well as fusion of host and viral membranes in viral diseases. Clearly, potential common regulators and mechanisms would be attractive therapeutic targets in these disorders.

Two of the cell types that fuse, osteoclasts and multinucleated giant cells, are derived from a common progenitor, are rendered fusion competent by common molecular mediators and ultimately regulate specialized functions in specific microenvironments. While OC can undergo fusion in both normal or pathological states such as Paget’s disease^33^, macrophages fuse to form MGC primarily under inflammatory conditions such as chronic granulomatous disease or the foreign body response^34^. However, the fact that the fusion of OC and MGCs are governed by common signaling mechanisms again makes these and their component molecules attractive targets for therapeutic intervention. Interestingly, we have previously shown that in response to ischemic injury, CD13^KO^ skeletal muscle satellite cells fused more readily than WT cells to form multinucleated myoblasts, suggesting that CD13 may also participate in fusion of other cell types, thus affecting processes such as skeletal muscle repair^16^.

Osteoclastogenesis comprises many steps from the commitment and survival of osteoclast progenitor cells, their differentiation into mononuclear pre-osteoclasts that fuse to generate multinucleated mature osteoclasts and finally, activation of osteoclasts for bone resorption. Among the different steps, osteoclast fusion is thought to be the critical step in this phenomenon. Our data clearly indicate that osteoclast progenitor survival, differentiation and proliferation is not dependent on CD13 expression, suggesting that CD13 may be specifically involved in the fusion mechanism to generate multinucleated osteoclasts.

Defective osteoblastic bone forming activity can also contribute to osteolysis. Previously, we have shown that CD13 expression does not affect mesenchymal stem cell formation or their survival^16^, confirming that elevated levels of osteoprogenitors in the in vitro osteoblastogenesis analysis is not due to differences in mesenchymal stem cell formation. We showed that despite an increase in the osteoblast progenitor population in the absence of CD13, bone formation rate and mineral apposition rate remain unaltered between genotypes, indicating that impaired skeletal mass is not due to a defect in mature osteoblast function. While dynamic bone formation is equivalent between genotypes of young mice, CD13^KO^ aged mice have a significantly reduced bone formation rate compared to their wildtype counterparts, perhaps implicating a CD13 contribution of osteoblast function in bone remodeling during aging.

Studies have implicated various membrane-associated processes as critical to osteoclastogenesis such as clustering of membrane tetraspanins^35^, endocytosis of surface molecules^36^ as well as the initial increases in expression of specific fusogenic molecules, such as DC-STAMP^29^, OC-STAMP^37^, dynamin and ATP6V0d2 (ATPase, H + transporting V0 subunit d2) and subsequent decline as osteoclastogenesis proceeds^36,38^. We see sustained expression of DC-STAMP, CD9 and dynamin-2 in CD13^KO^ cells while these proteins disappear in parallel WT cultures, suggesting that CD13 regulates fusion by mechanisms leading to decreased fusogen stability and the absence of CD13 results in sustained fusogen expression and enhanced fusion. Indeed, myeloid cells sorted for high levels of DC-STAMP expression formed higher numbers of OCs in fusion cultures than DC-STAMP low populations^39^. In agreement with these observations, transgenic mice overexpressing DC-STAMP driven by the actin promoter exhibited an osteoporotic phenotype and osteoclast hyper-multinucleation^11^, suggesting that sustained expression leads to bone loss. Clinically, Paget’s disease of the bone (PDB) is a disorder resulting from osteoclast overactivity of unknown etiology. Osteoclast-like cells isolated from PDB patients carrying a DC-STAMP genetic variant showed higher DC-STAMP expression levels and greater numbers of nuclei per cell compared to both healthy controls and PDB patients without the variant^40^. A distinct gain-of-function DC-STAMP variant (rs2458413) studied in cells from 158 patients was significantly associated with PDB^41^. Clearly, sustained expression of DC-STAMP and other fusogens would promote osteoclastogenesis and likely underlies the effects of CD13 on bone remodeling.

We have shown that CD13 regulates dynamin- and clathrin-mediated endocytosis^21,22^, recycling of cell surface proteins^20^ and Src activation^42^, each of which have been shown to participate in OC fusion. In the current study, fusion protein expression in CD13^KO^ OCs is aberrantly sustained by a posttranscriptional mechanism. Alternatively, organizers of actin-based protrusions are also pivotal in myeloid cell as well as gamete fusion^43^, which depends on step-wise reorganization of the actin cytoskeleton, initiated by formation of “podosome-like” membrane protrusions in myeloid cells^44–48^. Importantly, overexpression of DC-STAMP generates cells with numerous cell protrusions and increased fusogenic capacity. Similarly, the abundant filopodia in OC precursors and OC in active fusion are significantly downregulated as OCs mature^39,47,49^. Recently, we have reported that CD13 is a critical signaling platform that links the plasma membrane to dynamic mediators of actin cytoskeletal assembly and rearrangement^20^. We propose that CD13 may regulate the expression of these endocytic and fusion regulatory proteins, perhaps by mediating their internalization, endocytic trafficking, recycling and/or localization. Whether CD13’s regulation of fusogen stability affects formation of membrane protrusions or the localization of fusion regulators to the site of cell–cell fusion is currently under investigation.

In conclusion, in the present study we demonstrate that CD13 expression controls osteoclastogenesis specifically at the level of cell–cell fusion. Further investigation into the relationship between CD13 and dynamin, DCST1 and CD9 and other regulators of osteoclast fusion such as OC-STAMP and the osteoclast receptor αvβ3 integrin^28^ will clarify mechanisms regulating CD13-mediated cell fusion in osteoclasts as well as in fusion of other cell lineages including foreign body giant cells and satellite stem cells. Considering the diversity and importance of pathologies that are influenced by cell–cell fusion, identification of CD13-dependent molecular mechanisms and signaling that regulate myeloid fusion will provide novel therapeutic approaches in fusion pathologies.

## Materials and methods

The authors confirm that all experiments were carried out in accordance with relevant guidelines and regulations. The ethical approval for all animal care and procedures were carried out in accordance with relevant guidelines and regulations by the UConn Health Institutional Animal Care and Use Committee.

### Animals

Global young (8–10 weeks) or aged (18–25 weeks) wildtype and CD13^KO^ (C57BL/6J) male and female mice were generated and housed at the Gene Targeting and Transgenic Facility at University of Connecticut School of Medicine^12^. All procedures were performed in accordance with the guidelines and regulations approved by the UCONN Health Institutional Animal Care and Use Committee. UConn Health is fully accredited by Association for Assessment and Accreditation of Laboratory Animal Care (AAALAC) International and Public Health Service (PHS) assurance number is A3471-01 (D16-00295) and USDA Registration Number is 16-R-0025.

Euthanasia by CO2 followed by cervical dislocation was performed and is an accepted method consistent with AVMA Guidelines for the Euthanasia of Animals to minimize the pain or discomfort in animals.

### Reagents

Recombinant mouse and human M-CSF and RANKL were purchased from R&D Systems. Phorbol 12-myristate 13-acetate (PMA), Phosphatase, Leukocyte (TRAP) Kit were purchased from Sigma-Aldrich. Bovine cortical bone slices were a kind gift from Dr. Joseph Lorenzo, University of Connecticut School of Medicine^50^. Osteoplates were purchased from Corning.

### Antibodies

Antibodies to DC-STAMP1 (DCST1; Biorbyt, orb2242, rabbit polyclonal Ab), Dynamin (Abcam, ab3457, rabbit polyclonal Ab), CD9 (Abcam, ab223052, rabbit polyclonal Ab), Phalloidin-TRITC (Sigma-Aldrich, P1951), CD13 (SL13, Millipore, MABC950, rat monoclonal Ab).

### Flow cytometry

Antibodies for phenotypic analyses and sorting by flow cytometry used are as follows^24^; anti-CD3 (145-2C11), anti-B220 (RA3-6B2), anti-NK1.1 (PK136), anti-CD11b (M1/70), anti-CD115 (AFS98), anti-Ly6C (Al-21). All antibodies were purchased from Biolegend, BD Biosciences, e-Biosciences. UV Blue Live dead dye was purchased from Life Technologies. Labeling of cells for flow cytometry and sorting was performed as described. Briefly, flow cytometry on live cells were performed to obtain OCP cells from BM expressing CD11b^lo^ CD115^hi^ Ly6G^+^ (CD3/B220/NK1.1)^−^^24^ using BD-FACS Aria (BD Biosciences) and data analyzed with BD FACS DIVA version 9.0 (https://www.bdbiosciences.com/en-us/instruments/research-instruments/research-software/flow-cytometry-acquisition/facsdiva-software) and FlowJo version 9.9 software (https://www.flowjo.com/). For flow sorting of OCP, 200 × 10^6^ BM cells isolated from four pooled WT or four pooled CD13^KO^ mice were run through FACSAriaII to obtain OCP (5–8% of total sorted BM cells) analyzed by FACSDiva.

Surface expression of fusion promoting proteins was performed with goat anti-rabbit dynamin2-Alexa 488 (ProSci; 61-336), rabbit anti-mouse DCST1-Alexa fluor 350 (Bioss Inc; bs-8250R-A350), and rat anti-mouse CD9-APC (Biolegend, Clone MZ3; 124811) using BD LSRII-A and analyzed by FlowJo version 9.9 software (https://www.flowjo.com/). Goat IgG-Alexa 488 or rabbit IgG-AF350 or rat IgG-APC was used as isotype control.

### In vivo analysis of WT and CD13^KO^ mice

#### Micro-computed tomography and histomorphometry

Samples were scanned in a density-calibrated µCT40 (Scanco Medical, Bassersdorf, Switzerland) in PBS at 8 µm^3^ resolution with the following settings: 55 kV, 145 µA, 300 ms integration, 1000 projections/rotation with Gaussian filtering. Analysis was performed following standard guidelines^51^. Briefly, femoral trabeculae were auto-contoured in a 120-slice region 1 mm proximal to the distal condyles with a lower threshold of 2485 Hounsfield units (HU), and femoral cortex was auto-contoured in an 80-slice region just distal to the third trochanter with a lower threshold of 4932HU^50,52^. All samples were scanned, reconstructed, and analyzed in a Scanco µCT40 running Evaluation Program V6.6 (http://www.scanco.ch/en/systems-solutions/software.html).

### Isolation of hematopoietic progenitor population from bone marrow and spleen

BM cells were obtained by flushing femur and tibia from WT or CD13^KO^ mice with 10 ml 1 × PBS and 2% heat inactivated FBS, followed by RBC lysis and filtering through 40 μm cell strainer (BD Biosciences). Total live cells counted with Countess Automated cell counter (Thermo Fisher Scientific) were stained with antibody cocktail at 4 °C. Cells from mouse spleen was obtained by gentle crushing the organ between frosted microscopic slides in cold 10 ml 1 × PBS and 2% heat inactivated FBS.

### Generation and culture of osteoclast progenitors from bone marrow or spleen

Cells from mouse BM or spleen were stained with Ab cocktail containing anti-(CD3, B220, NK1.1, CD115, Ly6C) Ab and subjected to single-cell sorting by BD FACS Aria^24^. Flow-sorted osteoclast progenitors isolated from BM [CD11b^lo^ CD115^hi^ Ly6G^+^ (CD3/B220/NK1.1)^−^] or spleen [CD11b^hi^ CD115^+^Ly6C^hi^ (CD3/B220/NK1.1)^−^] at a density of 5000–20,000 cells/well (BM) or 20,000–50,000 cells/well (spleen) were seeded in 96-well dish in α-MEM containing 10%FBS, 1% Penicillin–Streptomycin, 30 ng/ml M-CSF and 30 ng/ml RANKL at 37 degree C with 5% CO_2_ for 0-10d. Multi-nucleated osteoclasts were stained with Tartrate-Resistant Acid Phosphatase (TRAP) staining and assessed by counting cells with more than three nuclei. Average area of osteoclast was measured by ImageJ software (https://imagej.nih.gov/ij/).

### Histomorophometric analysis and TRAP staining^52^

#### Dynamic histomorphometry of bone

To evaluate dynamic histomorphometry, mice of 8–10 weeks and 25 weeks of age were injected with calcein 10 mg/kg (Sigma) seven days and alizarin complexone 30 mg/kg (Sigma) intraperitoneally two days before they were sacrificed. Seven-micrometer sections of femora were analyzed under a fluorescent microscope (Leica DMR). Blinded researcher marked bone tissue, single, and double labels in Osteomeasure software (OsteoMetrics, Decatur, USA) (https://www.osteometrics.com). The software then automatically calculated the mineral apposition rate (MAR) and bone formation rate (BFR).

### Tartrate resistant acid phosphatase (TRAP) staining for osteoclasts

Following 4 days fixation in 4% PFA, bone samples were placed in 30% sucrose O/N at 4 °C prior cryoembedding (Cryomatrix, Thermo Fisher Scientific). Sections were cut onto Japanese Cryotape (Cryofilm 2C, Section Lab, Japan) at 7 µm and cross-linked using Norland Optical Adhesive 61 (Norland Optical) onto glass slides. Bone sections were decalcified for 30 min at RT and osteoclasts were stained for TRAP using a commercial staining kit (Sigma, St-Louis, MO). The sections were rinsed with distilled water, counterstained with hematoxylin for 30 s and air-dried. Osteoclasts were identified as multinucleated cells placed adjacent to the bone surface. Metaphyseal regions of TRAP stained femora, 1 mm distally from the epiphyseal plate, were analyzed under the microscope (Leica DMR). Blinded researcher marked osteoclasts and bone surface in Osteomeasure software (Osteo-Metrics) (https://www.osteometrics.com). The software then automatically calculated the trabecular volume (BV/TV, %) and osteoclasts per bone surface (Ocs/BS).

Histochemical analysis of TRAP^+^ osteoclasts in bone sections were imaged with Zeiss fluorescence inverted microscope and analyzed by using Zeiss Zen 2.0 Pro blue edition software (https://www.zeiss.com/content/dam/Microscopy/Downloads/Pdf/FAQs/zen2-blue-edition_installation-guide.pdf).

Flow-sorted osteoclast progenitor cells derived from BM or spleen in α-MEM (GIBCO BRL) containing 10%FBS, 1% Penicillin–Streptomycin, 30 ng/ml M-CSF and 30 ng/ml RANKL grown on plastic or UV-sterilized, devitalized bovine cortical bone slices (placed in 96-well dishes), at a density of 50,000 cells/well for indicated time were fixed in 2.5% Glutaraldehyde and TRAP stained according to manufacturer’s instruction (Sigma) and analyzed by Zeiss Zen 2.0 Pro blue edition software (https://www.zeiss.com/content/dam/Microscopy/Downloads/Pdf/FAQs/zen2-blue-edition_installation-guide.pdf).

### Bone resorption assay^52^

Flow-sorted osteoclast progenitors derived from BM were seeded on Osteo Assay plate (Corning) at a density of 50,000 cells/well in α-MEM containing 10%FBS, 1% Penicillin–Streptomycin, 30 ng/ml M-CSF and 30 ng/ml RANKL for d10. Surface pit formation was measured by removing cells with 100 μl of 10% bleach solution at RT for 5 min. Wells were washed with deionized water and allowed to dry. Cluster of pits formed was imaged using a light microscope (Olympus Scientific), using Olympus cellSens Dimension V0118 software (Olympus Scientific) (https://www.olympus-lifescience.com/en/software/cellsens/) and the area of resorption was measured by ImageJ software (https://imagej.nih.gov/ij/).

### Generation of CD13^KO^ U937 cells by CRISPR-Cas9 gene editing

Oligos containing the guide RNA sequence for human CD13: 5′-CAGTGCGATGATTGTGCACA-3′; guide RNA for scrambled control: 5′-CAGTCGGGCGTCATCATGAT-3′ were cloned into lentiCRISPR v2 (addgene plasmid #52961). Packaging plasmids psPAX2 and pMD2.G (addgene plasmid #12260 and addgene plasmid #12259) were utilized to generate the lentivirus as described^20^ in presence of 2 ng/µl puromycin to select for lentiCRISPR integration. Multiple CD13^KO^ and scrambled control clones confirmed by immunoblot, IF and flow cytometry analysis were employed for OC generation.

### Isolation of TRAP^+^ osteoclast-like cells from human monocytic cell line U937

U937 cells expressing CD13 CRISPR or scrambled control^20^ were grown at a density of 100,000 cells/well in 4-well dish with RPMI 1640 containing 10% heat inactivated fetal bovine serum, 1 mM l-glutamine, 1 mM sodium pyruvate, 1% penicillin/ streptomycin and 0.1 µg/ml PMA for 3 days. Non-adherent cells were removed and adherent cells were stimulated with M-CSF (60 ng/ml) and RANKL (100 ng/ml) in RPMI medium without PMA for an additional 10 days. Cells were fixed in 2.5% glutaraldehyde and osteoclast-like cells identified by TRAP staining.

### Immunofluorescence and microscopy

Flow-sorted osteoclast progenitors were grown on glass coverslips that were previously coated with 5 µg/ml fibronectin for indicated time period. Cells were fixed in 4% paraformaldehyde (Electron Microscopy Sciences) at RT for 30 min, permeabilized with 0.1% Triton-X-100 in PBS at RT for 5 min. Cells were blocked with blocking buffer containing 5% goat or donkey serum/5% BSA/1 × PBS at RT for 1 h followed by incubation with primary Ab in blocking buffer at 4 °C for overnight. Cells were washed and treated with secondary Ab (1:1200) and DAPI (nuclear stain) in blocking buffer at RT for 1 h. Coverslips were mounted with ProLong Gold antifade mounting medium (Life Technologies), visualized at excitation wavelength of 488 nm (Alexa 488), 543 nm (Alexa 594 or TRITC) and 405 nm (DAPI) and imaged by Zeiss LSM 880 confocal fluorescence microscope and analyzed by using Zeiss Zen 2.0 Pro blue edition software (https://www.zeiss.com/content/dam/Microscopy/Downloads/Pdf/FAQs/zen2-blue-edition_installation-guide.pdf).

### Immunoblot analysis

Flow-sorted BM osteoclast progenitors grown in presence of M-CSF and RANKL for 48 h were LPC (lysophosphatidylcholine)-synchronized by treating with reversible fusion inhibitor LPC (100 µM) for 12 h followed by washing and growing cells in LPC-free medium for fusion to proceed for 0–72 h. Cell lysates were harvested in 1% NP40 lysis buffer containing 1× complete Protease Inhibitor cocktail (Roche). Samples were separated by SDS-PAGE and transferred to nitrocellulose membrane, blocked in 1XTBST containing 5% bovine serum albumin, treated with primary Ab followed by appropriate secondary Ab and imaged by ChemiDoc Imaging system version 3.0.1 (https://www.bio-rad.com/en-us/category/chemidoc-imaging-systems?ID=NINJ0Z15) (Biorad). β actin or GAPDH were used as loading controls. Gels/blots were cropped and indicated by dividing lines.

### Quantitative RT-PCR analysis

Total RNA was extracted using TRIZOL reagent (Invitrogen) according to manufacturer’s instruction. Relative transcript level was normalized to GAPDH level. Primer sequences were determined using GenBank primer sequences (http://pga.mgh.harvard.edu/primerbank/). Sequence of PCR primers employed are as follows- Dynamin 2 (*Dnm2*), 5′-TTCGGGTCTACTCACCACAC-3′ (forward) and 5′-CTCTCGCGGCTGATGAACTG-3′ (reverse); DC-STAMP1 (*DCST1*), 5′-CGGCGGCCAATCTAAGGTC-3′ (forward) and 5′-CCCACCATGCCCTTGAACA-3′ (reverse); *CD9*, 5′-ATGCCGGTCAAAGGAGGTAG-3′ (forward) and 5′-GCCATAGTCCAATAGCAAGCA-3′ (reverse); *CD81*, 5′-CAGATCGCCAAGGATGTGAAG-3′ (forward) and 5′-GCCACAACAGTTGAGCGTCT-3′ (reverse); *GAPDH*, 5′-GGATTTGGTCGTATTGGG-3′ (forward), 5′-GGAAGATGGTGATGGGATT-3′ (reverse). All data was analyzed using CFX Manager version 3.1 (https://www.bio-rad.com/en-us/sku/1845000-cfx-manager-software?ID=1845000) (Biorad).

### Statistical analysis

Statistical analysis was performed using unpaired, two-tailed Student’s t test using GraphPad Prism software and results are representative of mean ± SD or ± SEM as indicated. Differences at p ≤ 0.05 were considered significant.

### Disclosures

All animal experiments in this study were reviewed and approved by Animal Care Committee at University of Connecticut Medical School.

## Supplementary Information

Supplementary Information.
