# Supplementary material for: CD13 is a critical regulator of cell–cell fusion in osteoclastogenesis
Source: Sci Rep. 2021 May 24;11:10736. doi: 10.1038/s41598-021-90271-x (PMC8144195; doi:10.1038/s41598-021-90271-x)
Supplement: Supplementary file 1 — Supplementary Information. [file 41598_2021_90271_MOESM1_ESM.pdf]

CD13 is a Critical Regulator of Cell-cell Fusion in Osteoclastogenesis

Mallika Ghosh[1*], Tomislav Kelava[2], Ivana Vrhovac Madunic[2], Ivo Kalajzic[2] and Linda H Shapiro,[1]

Centers for [1]Vascular Biology, [2]Regenerative Medicine and Skeletal Development
University of Connecticut Medical School, Farmington, Connecticut 06032, USA

***Corresponding authors**:

Linda H Shapiro, PhD
Center for Vascular Biology
University of Connecticut Medical School
Farmington, CT 06030
Email: lshapiro@uchc.edu
Telephone: 860-679-4373
Fax: 860-679-1201

Mallika Ghosh, PhD
Center for Vascular Biology
University of Connecticut Medical School
Farmington, CT 06030
Email: mghosh@uchc.edu
Telephone: 860-679-1189
Fax: 860-679-1201

Conflict of interest: The authors have declared that no conflict of interest exists.

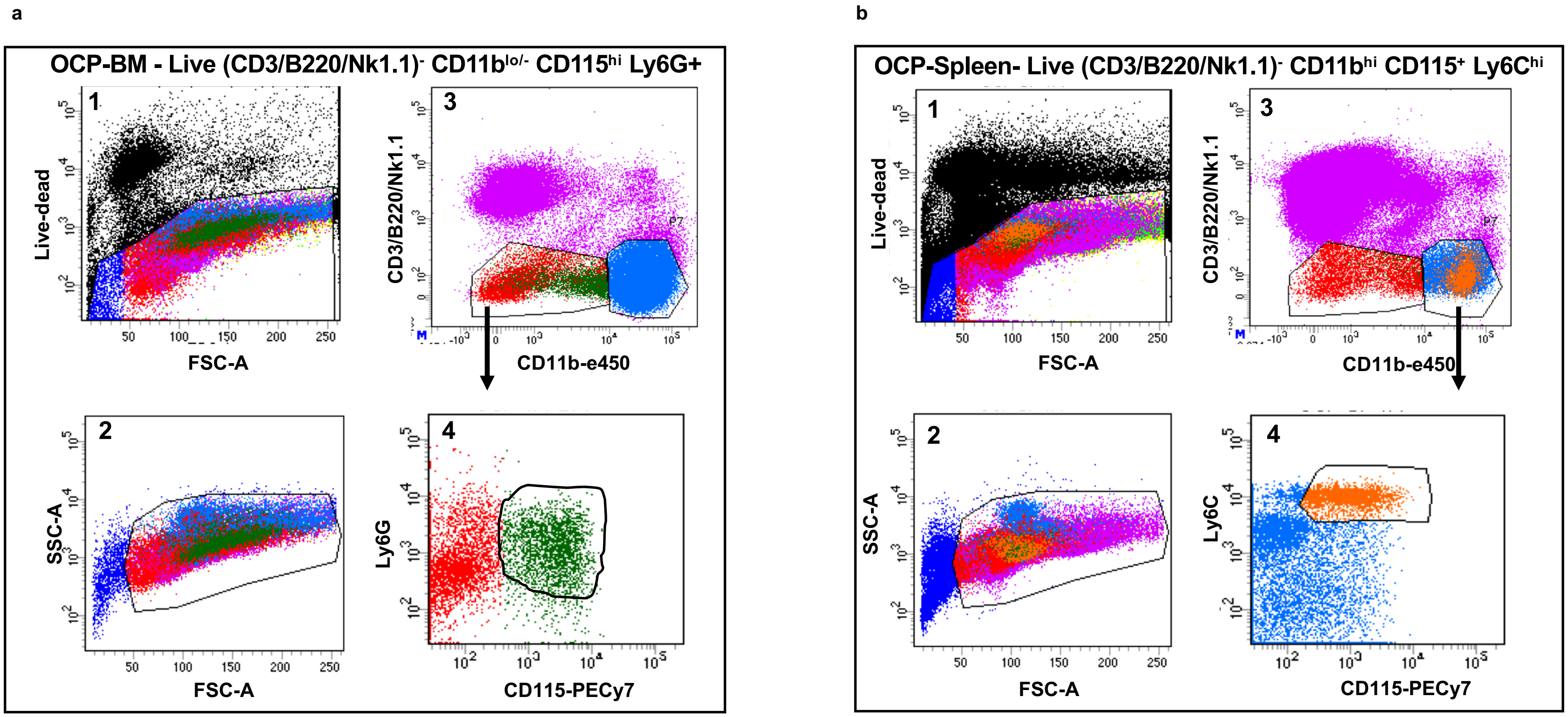

FIG S1. Representative image of flow sorting strategy of isolation of osteoclast progenitors from primary murine bone marrow or spleen from WT and CD13^{KO} mice. Live (CD3/B220/Nk1.1)$^-$ CD11b$^{lo/-}$ CD115^{hi} Ly6G$^+$ OCP cells were sorted from BM (**a**) or Live (CD3/B220/Nk1.1)$^-$ CD11b^{hi} CD115$^+$ Ly6C^{hi} OCP cells were sorted from spleen (**b**) to homogeneity using BD FACS ARIA and analyzed by FACS Diva version 9 (https://www.bdbiosciences.com/en-us/instruments/research-instruments/research-software/flow-cytometry-acquisition/facsdiva-software) and FlowJo (https://www.flowjo.com/) version 9.9.

FIG. S1

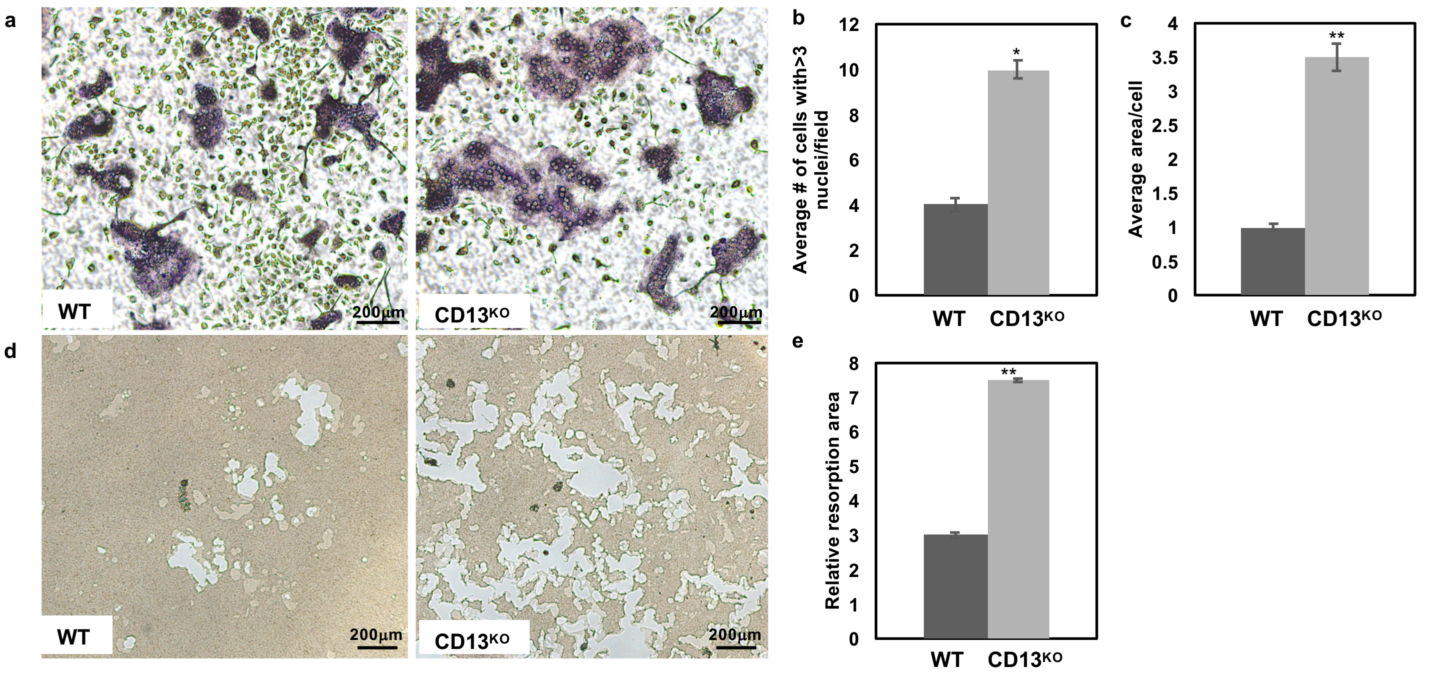

FIG S2. Formation of TRAP+ multinucleated BM-derived OCs isolated from aged mice are markedly enhanced in the absence of CD13. **a).** Primary murine BM derived OCs number and size /OC grown on tissue culture dish isolated from $CD13^{KO}$ and WT at d5. **b**). Average # of cells with >3 nuclei/field and **c**). Average cell area per OCs in $CD13^{KO}$ are significantly larger than the WT Ocs analyzed by and analyzed by using Zeiss Zen 2.0 Pro blue edition software (https://www.zeiss.com/content/dam/Microscopy/Downloads/Pdf/FAQs/zen2-blue-edition_installation-guide.pdf) . **d**). Area of resorption in $CD13^{KO}$ and WT OCs grown on osteoplates for d10 by phase contrast imaging using Olympus cellSens Dimension V0118 software (Olympus Scientific) (https://www.olympus-lifescience.com/en/software/cellsens/) & quantified by Image J (https://imagej.nih.gov/ij/) (**e**) are shown. Scale bar; 200µm. Data represents +/- SEM of three independent experiments. N=6/genotype, **;p<0.01, *;p<0.05.

FIG. S2

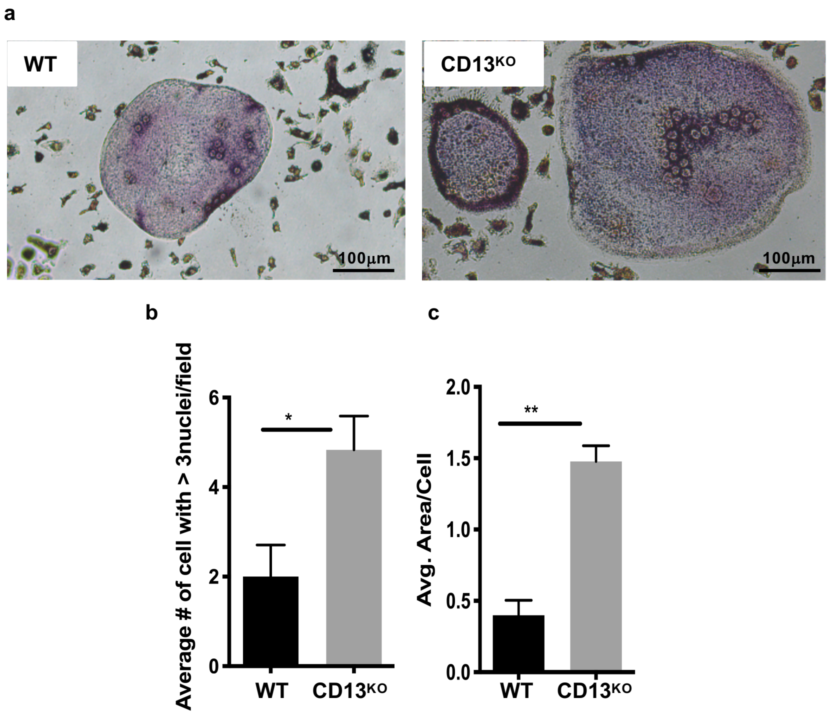

FIG S3. Increased TRAP+ splenic multinucleated OCs in the absence of CD13 in vitro. a). Flow-sorted primary murine spleen-derived OCP grown on plastic in the presence of M-CSF and RANKL led to enhanced OCs size and number of nuclei/OC in CD13^{KO} cells compared to WT at d7. **b**). Average # of cells with >3 nuclei/field and **c**). Average cell area per OC in CD13^{KO} are significantly larger than the WT cells analyzed by using Zeiss Zen 2.0 Pro blue edition software (https://www.zeiss.com/content/dam/Microscopy/Downloads/Pdf/FAQs/zen2-blue-edition_installation-guide.pdf) . Scale bar; 100μm. Data represents +/- SEM of three independent experiments. N=6/genotype, **;p<0.01.

FIG. S3

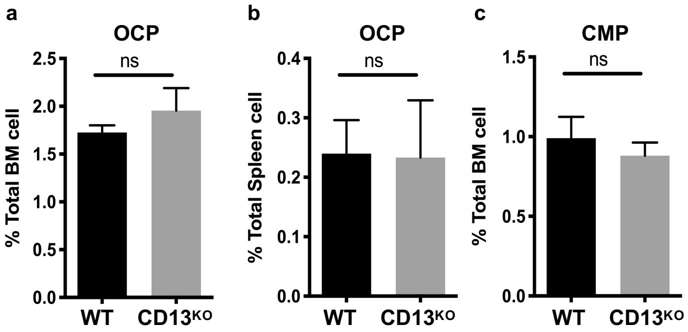

FIG S4. Osteoclast progenitors with osteoclastogenic potential are similar in WT and CD13^{KO} BM and periphery. Flow cytometric analysis of OCP profile of WT and CD13^{KO} mice indicated by CD3^{-}, B220^{-}, NK1.1^{-}, CD11b$^{-/lo}$, CD115^{+}, CD117^{+} in the BM (**a**), and CD3^{-}, B220^{-}, NK1.1^{-}, CD11b^{+}, Ly6G^{-}, Ly6C^{+}, CD115^{+} in spleen (**b)**, and common myeloid progenitor population indicated by lin^{-} c-kit^{+} Sca-1^{-} CD34^{+} in the BM analyzed by FlowJo software version 9.9 (https://www.flowjo.com/) (**c**). Data represents +/- SEM of three independent experiments. N=6/genotype.

FIG. S4

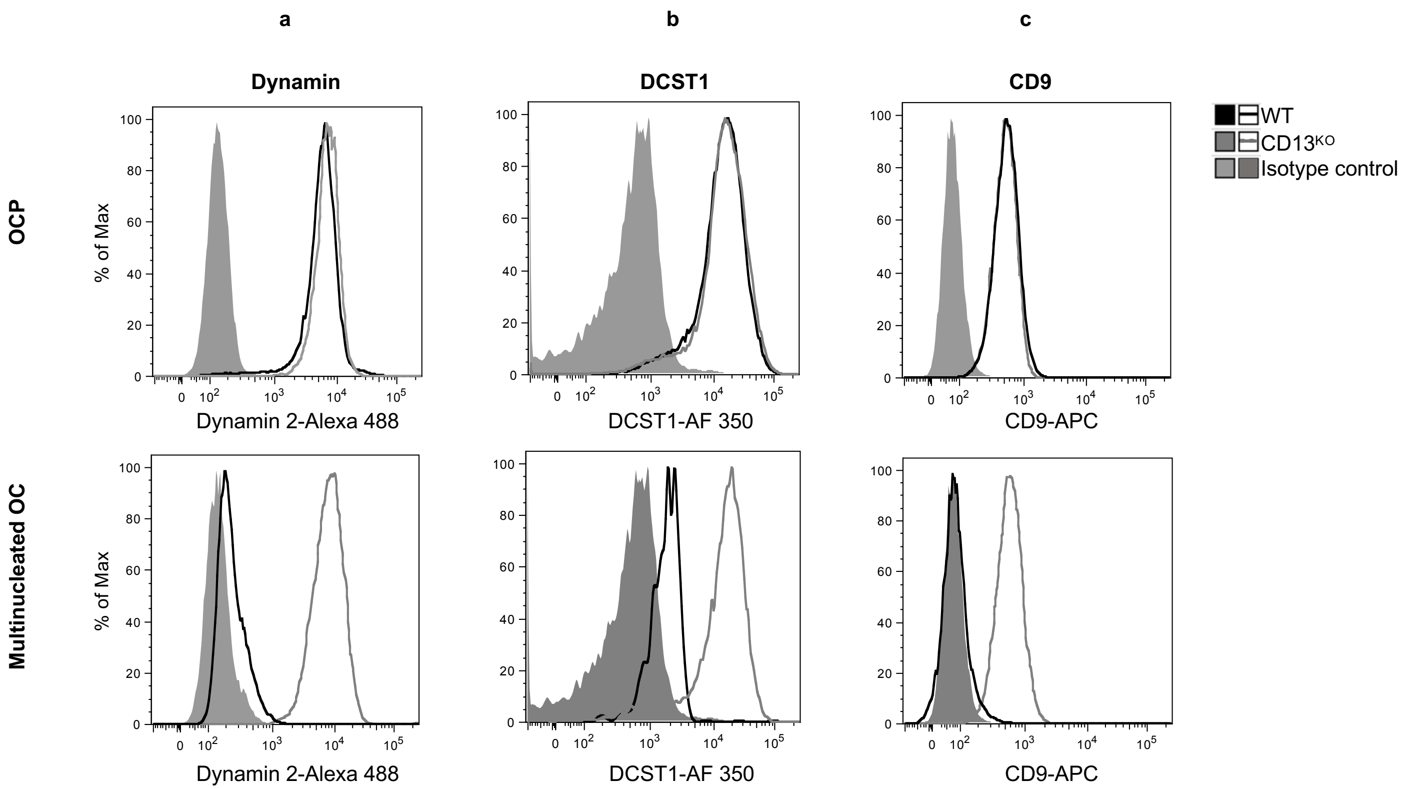

FIG S5. Representative histogram of dynamin, DCST1 and CD9 surface expression in osteoclast progenitors (OCP) and multinucleated OC isolated from WT and CD13^{KO} mice. Abundance at the surface was analyzed by staining with goat anti-rabbit dynamin2-Alexa 488 (**a**), rabbit anti-mouse DCST1-Alexa fluor 350 (**b**) and rat anti-mouse CD9-APC (**c**) followed by flow cytometry and analyzed by FlowJo software version 9.9 (https://www.flowjo.com/). Goat IgG-Alexa 488 (**a**) or rabbit IgG–AF350 (**b**) or rat IgG APC (**c**) was used as isotype control. N=3/genotype.

FIG. S5

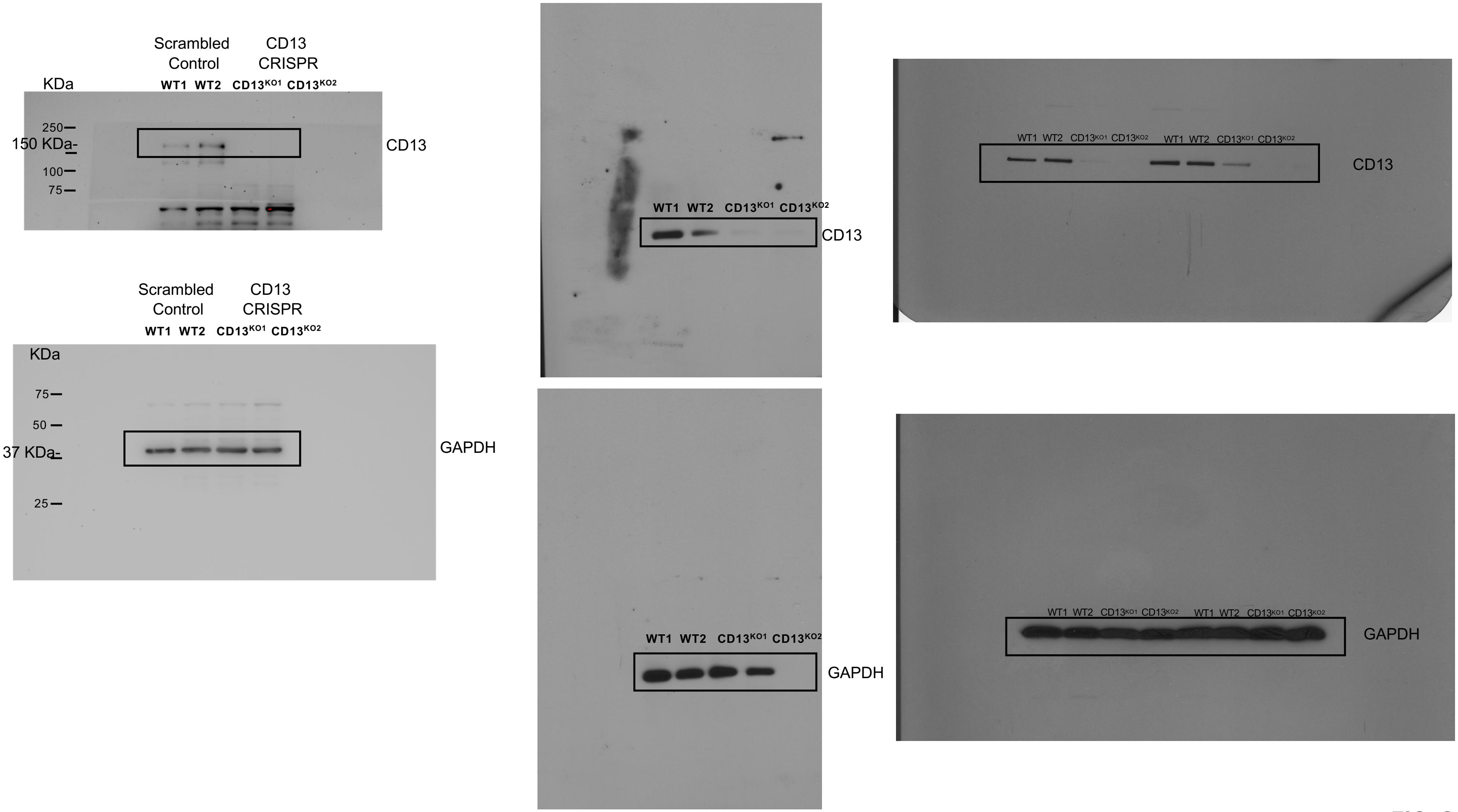

FIG. S6

FIG S6. Full-length individual blots and cropped replicates of Fig. 5f.

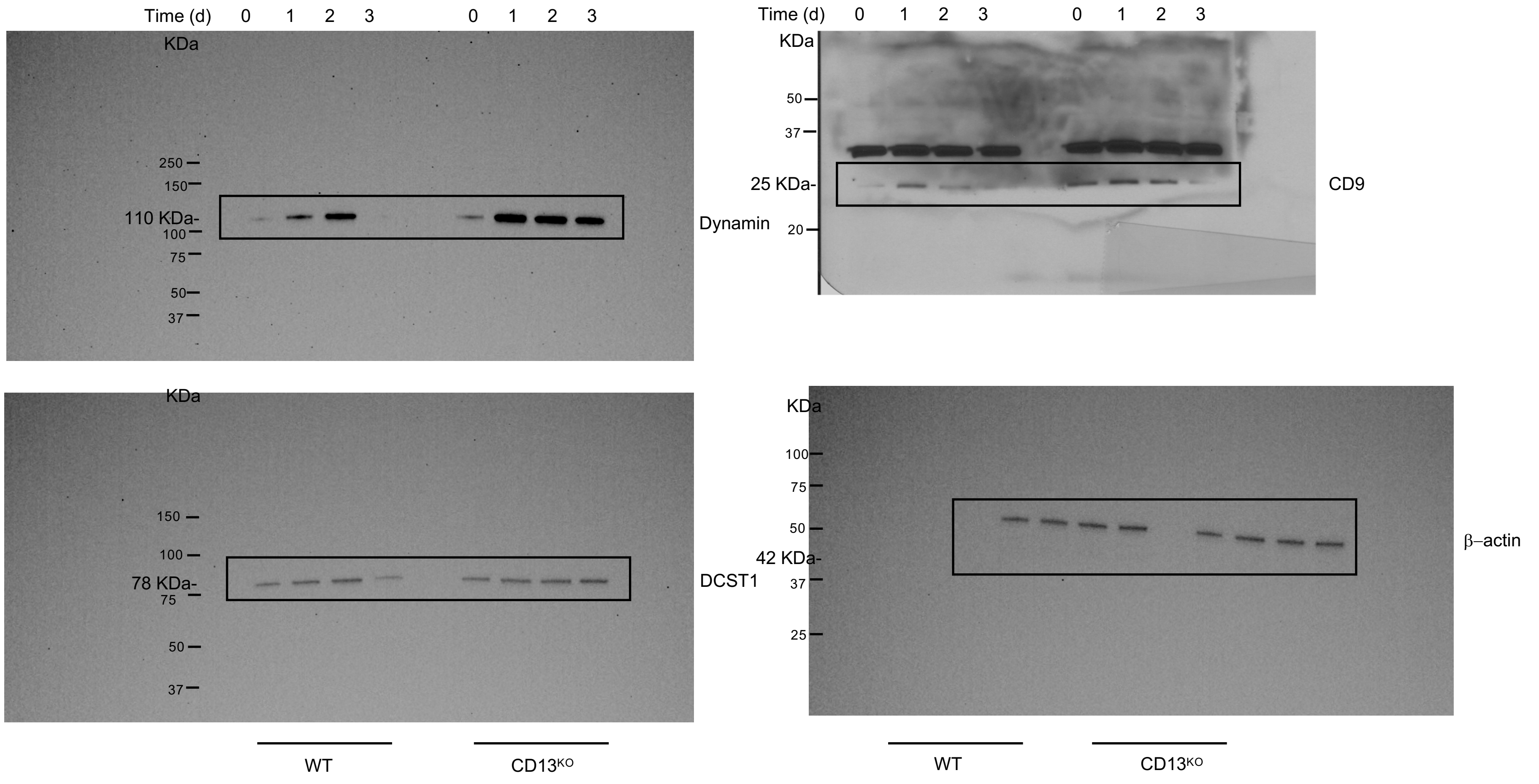

FIG S7. Full-length individual blots of Fig. 7a.

FIG. S7

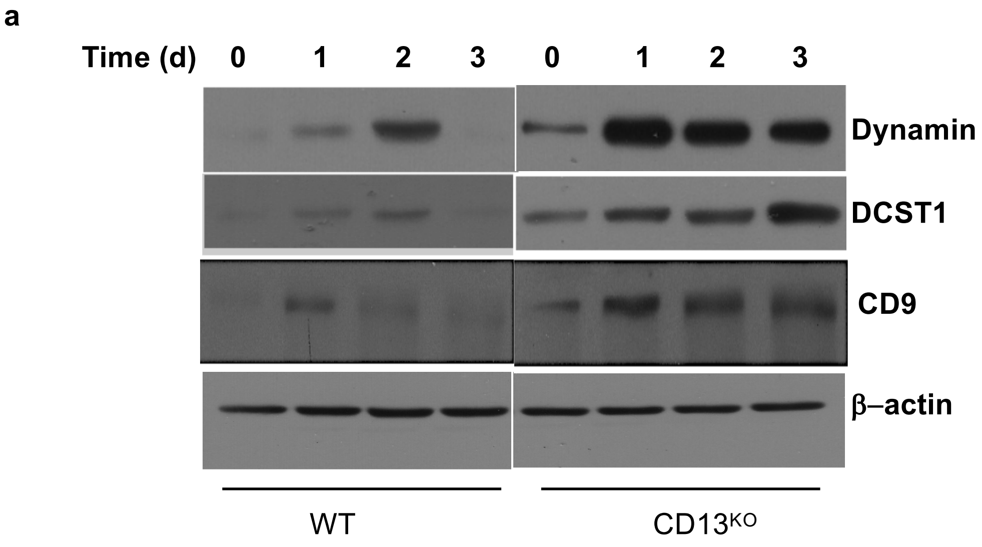

FIG S8. Cropped replicates of Fig. 7a.

FIG. S8

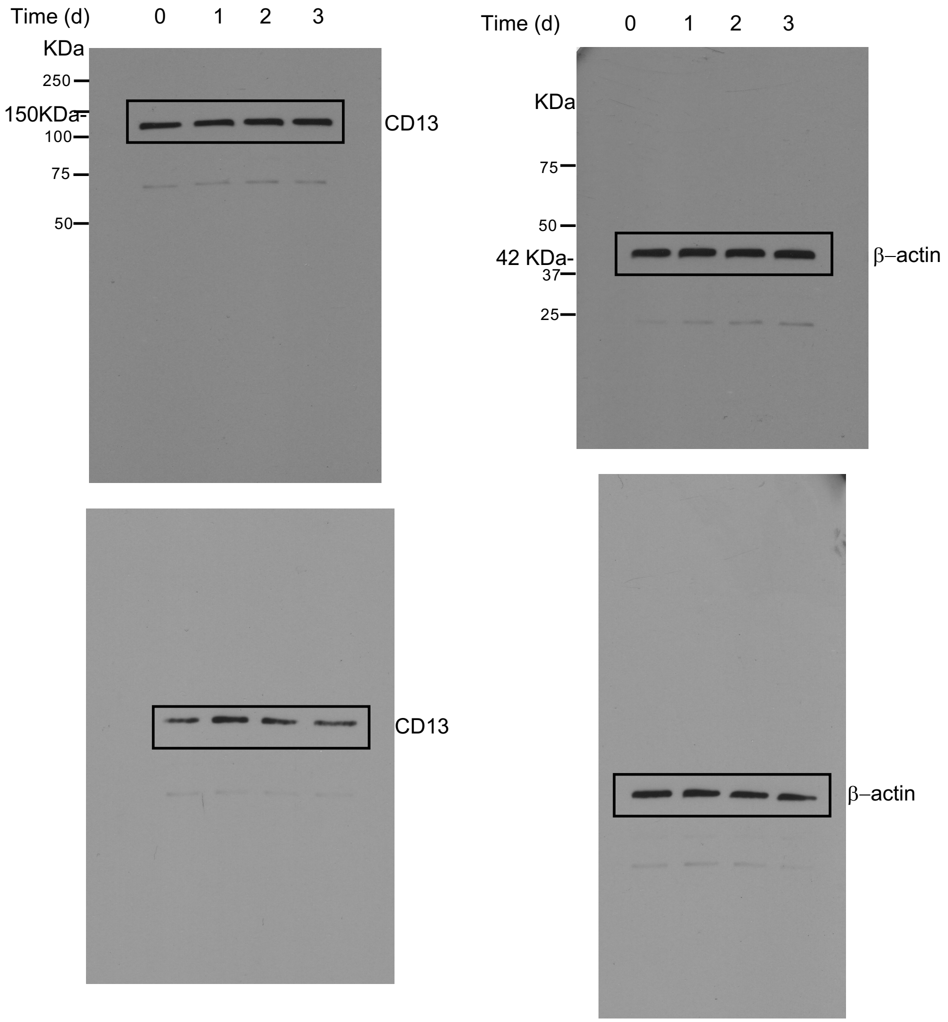

FIG S9. Full-length individual blots of Fig. 7c.

FIG. S9

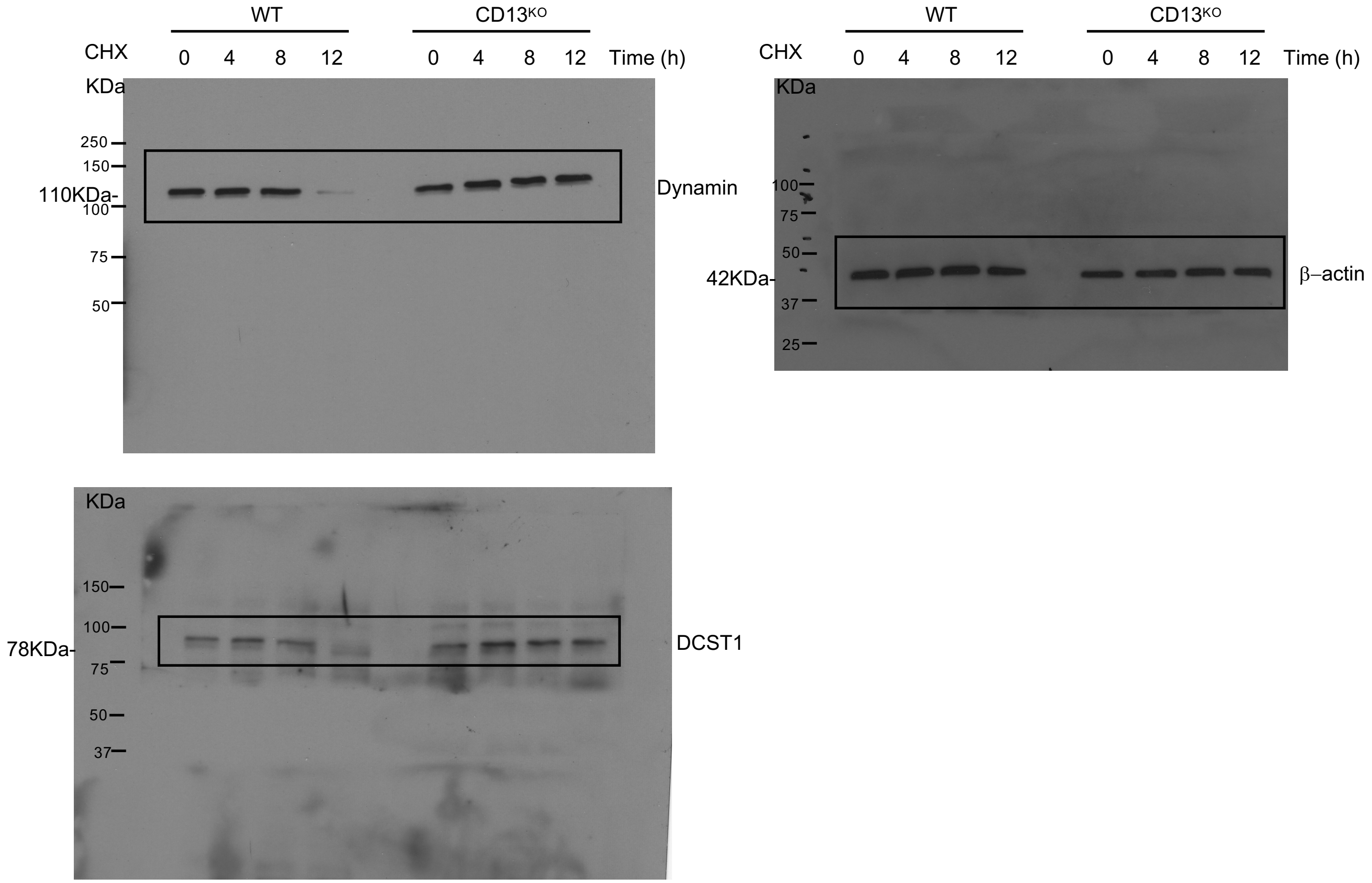

FIG S10. Full-length individual blots of Fig. 8b.

FIG. S10

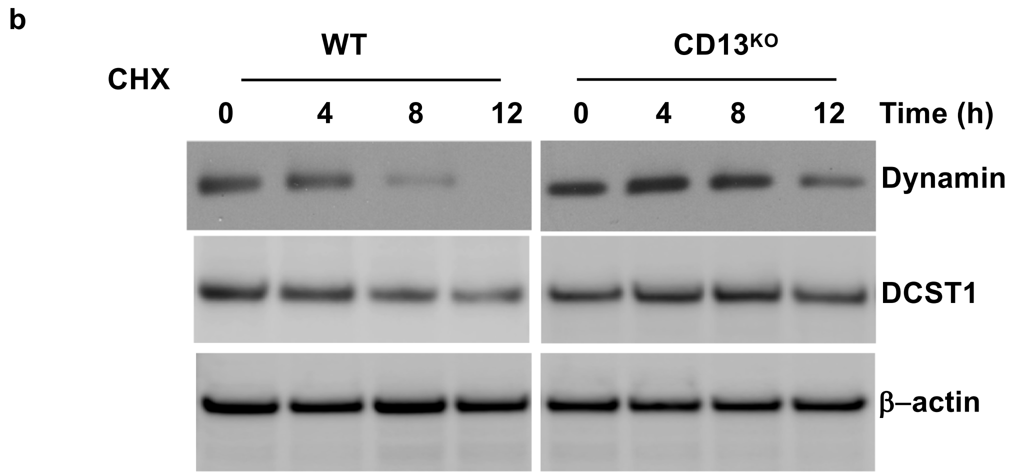

FIG S11. Cropped replicates of Fig. 8b.

FIG. S11
